# Small-area spatio-temporal analysis of cancer risk to support effective and equitable cancer prevention

**DOI:** 10.1371/journal.pone.0325523

**Published:** 2025-06-09

**Authors:** Nathalie Saint-Jacques, Judy Purcell, Patrick E. Brown, Daniel G. Rainham, Trevor J. B. Dummer

**Affiliations:** 1 Nova Scotia Health Cancer Care Program, Halifax, Nova Scotia, Canada; 2 Department of Medicine, Dalhousie University, Halifax, Nova Scotia, Canada; 3 Healthy Populations Institute, Dalhousie University, Halifax, Nova Scotia, Canada; 4 Department of Statistical Science, University of Toronto, Toronto, Canada; 5 School of Health and Human Performance, Dalhousie University, Halifax, Nova Scotia, Canada; 6 School of Population and Public Health, University of British Columbia, Vancouver, British Columbia, Canada; The University of Sydney, AUSTRALIA

## Abstract

Cancer is rapidly increasing worldwide and urgent global action towards cancer control is required. Consistent with global trends, Canada is expected to experience a near doubling in new cases and cancer deaths between 2020–2040; population growth and ageing being the primary drivers. The projected increased cancer incidence and its associated costs is expected to further exacerbate socioeconomic inequities. Focused actions to prevent cancer, to detect it earlier when more treatable, and, to lower the risk of recurrence, must be prioritized. Almost half of all cancers are preventable, caused by risk factors that are potentially avoidable and modifiable. Integrating cancer prevention with care-based models is necessary and represents the most cost-effective and sustainable approach to control cancer. To be effective, prevention efforts must consider the cancers impacting local populations and understand how community and individual factors interact within the spatial and temporal contexts in which people live. This study is part of the Nova Scotia Community Cancer Matrix project which profiles the cancers impacting communities over time; measuring associations between cancer and socioeconomic status (SES); and determining how the joint spatial distribution of cancers can be used to address inequities, identify priority populations and strengthen prevention efforts. Using Bayesian inference to model spatio-temporal variations in 58,206 cases diagnosed in 301 communities between 2001–2017, across 10 preventable cancer types, we report significant disparities in cancer risk across communities based on sex and community SES. The work highlights the utility of small-area mapping to identify at-risk communities and understand how community-SES impacts risk. It also uncovers significant inequities rooted in the differential distribution of material and social capacity, operating beyond the control of individuals. The approach is implementable to other regions to inform and strengthen prevention efforts aiming at reducing the burden of cancer or that of other diseases.

## Introduction

The global burden of cancer is increasing worldwide, highlighting an urgent need for investments and actions towards cancer control. In 2020, an estimated 19.3 million new cancer cases and 10 million cancer deaths were reported globally [1]. By 2040, the global cancer burden is expected to rise 47%, to 28.4 million cases annually. These trends are expected to continue as countries undergo varying levels of demographic and epidemiological transition, where ageing populations and chronic non-communicable disease become more dominant, and where existing and emerging risk factors and exposures remain unaddressed [2].

In high-income countries such as Canada, cancer has become the leading cause of premature death [2,3]. Currently, 45% of all people in Canada receive a cancer diagnosis in their lifetime and one in four dies from the disease. Consistent with the global trends, Canada is predicted to experience a 40% rise in new cancer cases and a 44% increase in cancer deaths during the period 2020–2040 [4]. Population ageing and growth are the primary factors leading the progressive and significant increase in the projected number of cancer cases [5]. The estimated $31.4 B (CAD) needed to manage the impact of cancer in Canada in 20 years [4] will further burden an already overextended healthcare system.

The projected increase in cancer incidence and associated healthcare costs will also further exacerbate health inequities. In Canada, despite having a largely publicly funded health care system, almost 30% of the costs associated with a cancer diagnosis are borne by patients and their families, many of whom report financial distress [6,7]. People who are socially disadvantaged experience these costs disproportionately, jeopardizing their ability to access health services, timely diagnosis and treatment— issues which lead to less favourable outcomes [8,9]. Ultimately, this creates a feedback loop that reinforces marginalization and inequities at all points of the cancer continuum.

Actions to tackle these challenges are urgently needed for effective and equitable cancer control. Imbalances in resources distribution and access to care calls for investments beyond the development and adoption of resource-intensive interventions to which many patients lack access. Advances in cancer treatment alone will not solve the global cancer crisis [2,10,11]. Focused actions to prevent cancer from developing, to detect cancer earlier when it is more treatable, and to lower the risk of future recurrence, must be prioritized. A growing body of knowledge has demonstrated that almost half of all cancers are preventable because cancer is often caused by risk factors that are avoidable, modifiable, and actionable [12,13].

Integrating preventive measures to care-based models is necessary and, possibly, the most cost-effective and sustainable intervention for cancer control [14,15]. However, implementing preventive measures requires an in-depth understanding of the cancer burden impacting local populations, as well as the risk factors—past and future—that may act independently, simultaneously, or synergistically to impact cancer outcomes. Therefore, effective, cancer prevention efforts must consider both the individual person-level characteristics and the spatial context in which the cancer develops. Neighbourhoods, provide the context of the physical and social attributes that can affect the health of individuals [16]. Therefore, being able to express patterns of cancer co-occurrence using a geographically focused approach, can help identify underlying mechanisms for targeted prevention and to identify communities that require additional support.

This study profiles spatio-temporal heterogeneity in cancer risk across communities in Nova Scotia, Canada and demonstrates the utility of small-area analysis to support cancer control programs and interventions that can more effectively address inequity in risk distribution and target priority populations. The work is part of the Nova Scotia Community Cancer Matrix (NS-Matrix), described in more detail elsewhere [17,18]. In this analysis we focus on 10 preventable cancers and the associations between cancer risk and socioeconomic status (SES); and determine how the joint spatial distribution in the risk of these cancers can be used for targeted prevention and health promotion.

## Materials and methods

### Data sources

Nova Scotia (NS) is a province in Eastern Canada with a population of one million. It has the second to lowest gross income per capita in Canada and experiences some of the highest rates of cancer, and prevalence of cancer risk factors (e.g., smoking, alcohol consumption, physical inactivity, food insecurity, people living with obesity), in the country. The study cohort included 58,206 invasive first primary cancers diagnosed in NS residents over the period of 01/01/2001–31/12/2017, across 10 preventable cancer types: bladder– including in situ (bladder; C67), female breast (breast; C50), cervix uteri (cervix; C53), colorectal (C18–C20, C26.0), head and neck (C00–C14, C30-C32.9), liver (C22.0, C22.1), lung and bronchus (lung; C34), melanoma of the skin (melanoma; C44 (type 8720–8790)), pancreas (C25), and stomach (C16). Case selection followed the multiple primary rules established by the International Agency or Research on Cancer, and disease coding followed the standards of the World Health Organization International Classification of Disease for Oncology (ICD-O). Cases were aggregated over four time periods 2001 (2001–03), 2006 (2004–08), 2011 (2009–13), and 2016 (2014–2017) to increase numerical stability. Data, including, sex at birth, age at diagnosis and spatial location at time of diagnosis, were accessed from the NS Cancer Registry for research purposes on the 15/10/2019 following an application to the NS Health (NSH) Data Access Committee and Research Ethics Board (REB # 1023913). All cases were georeferenced to one of 301 Community Environs (COMe) using residential civic address at time of diagnosis. Where complete address was unavailable, spatial coordinates were obtained from six-digit postal codes using the Postal Code Conversion File (PCCF+) which assigns a postal codes to dissemination area (DA) based a on population-weighted approach. DAs are the smallest unit of geography for which Statistics Canada provides coordinates. In NS, the population of DAs vary between 50–6,000 people. Details relating to the creation of the COMe geography can be found in Saint-Jacques et al. 2023 [17]. All base maps were obtained from Statistics Canada [19].

**Fig 1:**
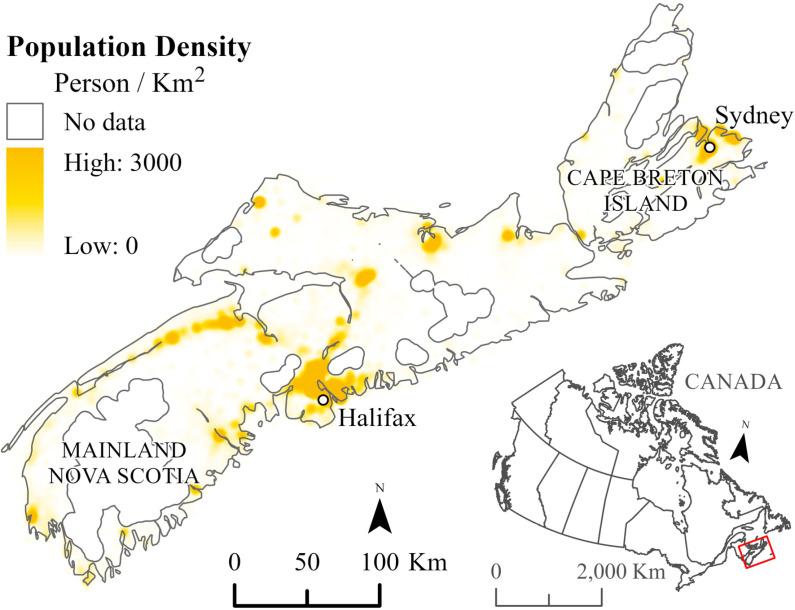
Population density, Nova Scotia, Canada. Base Map Source: Statistics Canada, Census Dissemination Areas Boundary File, 17 Nov 2021. Reproduced and distributed on an “as is” basis with the permission of Statistics Canada [19].

Population counts from semi-custom files aggregating population from dissemination-block to COMe were obtained from Statistics Canada census 2001, 2006, 2011 and 2016. The data were used to determine population at risk by sex and age for each of the four time periods. Communities with at least 1,000 people and a population density of 400 + persons per km^2^, were considered urban, following Statistics Canada definition of urban population centres [20] and was used to adjust for the effect of urbanicity. Fig 1 shows how population density varies across NS and highlights the two largest population centres: Halifax and Sydney. Based on the latest 2021 census, these population centres hosted 439,819 and 30,960 people, respectively.

Area-based composite indices measuring material security (MS) and social connectivity (SC) were used to capture the independent effect of the contextual setting of a place which can impact health-related lifestyle behaviours [21–24]. The indices, computed independently, are an adaption of the Canadian ‘deprivation indices’ introduced by Pampalon and Raymond [25] but include a larger number of variables to account for sociodemographic diversity and the broader aspects of material wealth. In addition, given the indices were computed over small areas and four time periods, we applied a local conditional autoregressive model for spatially autocorrelated errors and applied a lag 1 autoregressive model for temporally related observations to the correlation matrices used for the calculation of scores [26]. The MS scores summarised the average correlational structure from nine census variables crossing *education* (% people with no high school diploma aged 65 + years or % with no post-secondary diploma aged 25–64 years), *income* (mean individual income, % people on low income after tax aged 24–65 years or those aged 65 + years, % people receiving government transfers, % people spending >30% income on shelter), *employment* (unemployment rate), and *housing* (dwelling average value, % people living in house in need of major repairs); scores for SC summarized the average correlational structure from seven census variables crossing domains of *social connection* (% people who are widowed, divorced or separated, % single-parent families, % people living alone, % people who have moved in past year) and *ethnicity* (% recent immigrant, % visible minority, % people who speak no official languages). The COMe geographic-level scores were ranked and grouped into weighted population quintiles, representing high (Q1) to low (Q5) SES communities. All variables were derived from Statistics Canada semi-custom census profiles (2001, 2006, 2011, 2016), aggregated to COMe from dissemination block values.

### Data analysis

Spatial and spatio-temporal variations of cancer-specific relative risks (RRs) were modeled using a variation of a Conditional AutoRegressive (CAR) model, also commonly known as a Besag, York and Mollié (BYM) model [26–28]. For each cancer, case counts $Y_{st}$ observed at each COMe ‘s’ and time ‘t’ were treated as a Poisson process with a latent relative risk $\lambda_{st}$ and offsets of expected counts $E_{st}$, derived from the age-specific incidence rates of NS applied to the age-sex composition found in each COMe and time. Here, ‘~’ indicates ‘distributed as’. A log-link was assumed, with log-linear fixed effects $\beta_{k}$ ,model matrix $X_{st}^{T}$, random spatial effects $U_{s}$ ,and random spatio-temporal effects $W_{st}$:


$$Y_{st}\sim Poisson\left(E_{st}\lambda_{st}\right)$$

$$\log\left(\lambda_{st}\right)=X_{st}^{T}\beta_{k}+U_{s}+W_{st}$$

$$U_{s}\sim BYM\left(\varphi_{U},\sigma_{U}^{2}\right)$$

$$W_{st}\sim AR1\left(\rho_{W}\right)⊗BYM\left(\varphi_{W},\sigma_{W}^{2}\right)$$

The spatial effects were assumed to follow a BYM distribution (Simpson et al.’s 2017 parameterization) [26] with the marginal variance $\sigma_{U}^{2}$ attributable to spatial processes (neighbourhood and unstructured) and $\varphi_{U}$ the proportion of this marginal variance attributable to neighbourhood effects. A separable model was used for the spatio-temporal effects, with the variance matrix being the Kronecker product of a BYM correlation and a first-order autoregression in time (AR1) with an autocorrelation parameter $\rho_{W}$.

Decomposing the extra Poisson variation in this way was necessary as several cancers were expected to show spatial and spatio-temporal variability; this consequently permits more precise estimation of the excess risk attributable to measured covariates. Further, as the BYM borrows information from neighbouring areas, it provides more stable parameter estimates. Similarly, time variations in the spatio-temporal random effect being modeled as an AR1 process also helped stabilize RRs associated with strong temporal variability. Integrated Nested Laplace Approximation (INLA) was used for Bayesian inference [28–30]. The parameters for the k = 11 fixed effects (intercept, time, MS, SC, urbanicity) were assigned the following priors. For the intercept, an uninformative prior with a mean of the overall median crude standardised incidence ratio (SIR) of a given cancer over the entire study period and variance 1000 was assigned. For the remaining fixed effects, a Normal prior with mean zero and a variance of 100 was assigned. Both priors were flat relative to posterior distributions, for all cancers considered. Priors for the random effects were as follows. The standard deviation of the spatial BYM was assigned an exponential prior with a median equal to the overall standard deviation of the crude standardised incidence ratios (SIRs) of a given cancer over the entire study period, and a PC-Prior [26] with a median of 0.5 was used for the spatial proportion. The autoregressive parameter of the spatio-temporal component had an exponential prior where the 90% quantile is 0.5, a fairly informative prior encouraging independence in time; the spatial proportion of the spatio-temporal component was assigned the same PC-prior as the spatial process. Posterior probabilities for exceedance were used to assess uncertainties associated with the modeled median RRs of the posterior predictions and spatial random effects. These probabilities provide a measure of confidence that the estimated community-specific risk is higher than that of the overall average for the study region and period. A probability threshold ≥ 80% (P_high_) suggests a RR above average, pointing to a high-risk area [31,32].

Patterns of joint spatial distribution/co-association of cancers for the most recent period (2014–2017) and that reflecting long-term, persistent excess risk and measured over the study period (2001–2017), were assessed using two area-based Composite Indices of Cancer (CIC) derived from Principal Component Analysis (PCA). A PCA is an eigen decomposition (rotation) of a symmetric matrix to an orthogonal (uncorrelated) set of new variables (i.e., principal components) where the first component summarizes most of the variability in the data. In this study, the first index, the ‘spatial’ CIC (sCIC), represented the first component from a PCA of the cancer-specific median RRs of the spatial random effects [33,34]. The second index, the ‘predicted’ CIC (pCIC), focused upon the first component from a PCA of the cancer-specific median posterior predicted RRs for the 2016 period. Scores for both indices are a linear combination of the scaled RRs associated with each cancer type (8 in males; 10 in females) and are estimated in each community (COMe). Communities with similar scores will tend to have similar cancer profiles. Data analysis and processing was performed using R (version 4.3.1) [35], SAS software (version 9.4), and ArcPro (version 2.9.3).

## Results

### Cohort characteristics and models parameters

Analyses were based on 58,206 cancers diagnosed over the period of 2001 and 2017, across 10 cancer types (Table 1). Lung and colorectal cancers were most common, accounting for 49% of all cases. The strongest sex-differences in case distribution were seen for bladder, head and neck, and liver cancers for which about 75% of the cases were diagnosed in males. Mean age at diagnosis was comparable across sexes, cancer types and time periods. The percentage of cases with assigned spatial location based on complete civic residential address, increased from an average of 79.4% to 94.4% between 2001 and 2016. Overall, 89% of all cases were georeferenced using this most precise geolocation, the remaining were georeferenced using a six-digit postal code which represents a key element of postal address in Canada.

**Table 1:** Characteristics of study cohort.

Cancer type ^a^		Cases analysed ^b^			Mean age at diagnosis (years)		Spatial referencing (%)	
	Period	Total	Females	Males	Females	Males	Civic address	Postal code
**Lung**	2001	2,350	984	1,366	68.2	69.5	78.0	22.0
	2006	4,224	1,898	2,326	68.8	70.0	86.3	13.7
	2011	4,533	2,156	2,377	69.9	70.5	90.7	9.3
	2016	3,951	1,944	2,007	70.4	70.9	93.3	6.7
	**2001-2017**	**15,058**	**6,982**	**8,076**	**69.5**	**70.3**	**88.2**	**11.8**
**Colorectal**	2001	2,172	1,000	1,172	71.8	68.4	78.3	21.7
	2006	3,836	1,797	2,039	71.8	68.9	87.5	12.5
	2011	4,024	1,865	2,159	70.7	68.9	91.6	8.4
	2016	3,238	1,439	1,799	71.1	69.1	94.8	5.2
	**2001-2017**	**13,270**	**6,101**	**7,169**	**71.3**	**68.9**	**89.0**	**11.0**
**Breast**	2001	2,138	2,138	.	62.1	.	80.2	19.8
	2006	3,678	3,678	.	62.7	.	89.1	10.9
	2011	3,867	3,867		62.6	.	93.0	7.0
	2016	3,246	3,246		63.5	.	95.0	5.0
	**2001-2017**	**12,929**	**12,929**		**62.8**	.	**90.3**	**9.7**
**Bladder**	2001	694	184	510	70.8	70.0	78.2	21.8
	2006	1,212	305	907	71.8	71.0	88.1	11.9
	2011	1,411	379	1,032	70.7	71.3	92.0	8.0
	2016	1,252	307	945	71.7	71.7	94.4	5.6
	**2001-2017**	**4,569**	**1,175**	**3,394**	**71.3**	**71.1**	**89.5**	**10.5**
**Melanoma**	2001	534	263	271	57.0	61.4	77.3	22.7
	2006	1,015	494	521	59.4	61.2	87.4	12.6
	2011	1,248	621	627	59.4	63.6	90.5	9.5
	2016	1,259	567	692	61.1	64.5	94.7	5.3
	**2001-2017**	**4,056**	**1,945**	**2,111**	**59.6**	**63.0**	**89.3**	**10.7**
**Head and**	2001	495	127	368	64.2	64.0	78.0	22.0
**neck**	2006	774	201	573	65.3	62.9	85.4	14.6
	2011	879	244	635	67.8	63.8	88.7	11.3
	2016	782	207	575	65.9	64.6	93.9	6.1
	**2001-2017**	**2,930**	**779**	**2,151**	**66.1**	**63.8**	**87.4**	**12.6**
**Pancreas**	2001	338	162	176	73.7	69.4	79.6	20.4
	2006	662	344	318	73.3	69.8	87.5	12.5
	2011	693	347	346	72.2	68.8	92.9	7.1
	2016	668	312	356	73.6	69.9	95.8	4.2
	**2001-2017**	**2,361**	**1,165**	**1,196**	**73.1**	**69.5**	**90.3**	**9.7**
**Stomach**	2001	281	95	186	71.9	68.4	77.2	22.8
	2006	493	168	325	72.0	68.2	84.4	15.6
	2011	428	142	286	72.0	68.6	92.1	7.9
	2016	356	129	227	73.2	69.9	94.9	5.1
	**2001-2017**	**1,558**	**534**	**1,024**	**72.3**	**68.7**	**87.6**	**12.4**
**Cervix**	2001	166	166	.	48.3	.	80.7	19.3
	2006	240	240	.	50.6	.	86.6	13.4
	2011	206	206	.	51.5	.	94.2	5.8
	2016	143	143	.	50.5	.	93.0	7.0
	**2001-2017**	**755**	**755**	.	**50.3**	.	**88.5**	**11.4**
**Liver**	2001	63	20	43	63.0	68.3	87.3	12.7
	2006	174	40	134	68.1	66.4	87.9	12.1
	2011	231	42	189	73.0	64.8	90.9	9.1
	2016	252	59	193	66.8	67.2	95.6	4.4
	**2001-2017**	**720**	**161**	**559**	**68.2**	**66.3**	**91.5**	**8.5**

^a^Sorted by frequency for period 2001–2017; ^b^ Excludes 9 cases due to unavailable spatial information.

Cancer-specific model parameters can be found in Table 2. Spatial processes were significant for all cancers considered (SD sp. CrIs > 0), showing the presence of important extra-Poisson variations. Overall, spatial (SD sp.) and spatio-temporal (SD sp.t.) processes had similar magnitude effects based on their respective overlapping credible intervals, except for lung cancer. For this cancer type, spatial processes dominated for both sexes. In males, neighbourhood effects and unstructured spatial effects were of similar magnitude (Phi sp. 0.56), whereas, in females, neighbourhood effects were more pronounced (Phi sp. 0.78). Liver, bladder and lung cancers demonstrated the largest neighbourhood effects in males (Phi sp 0.76, 0.63, 0.56, respectively). Lung cancer was also highly clustered in females along with cervical cancer (Phi sp. 0.78, 0.66, respectively). The temporal autocorrelation in the spatio-temporal processes (Group Rho) was non-significant for all cancers, suggesting that the main fixed effects accounted for the majority of the variation in cancer RR across time periods.

**Table 2:** Parameters and associated 95% credible intervals (CrIs) from the spatio-temporal models.

Cancer type	Parameter	Males			Females		
		Median	2.5%	97.5%	Median	2.5%	97.5%
**Bladder**	Phi sp.	0.63	0.14	0.96	0.43	0.14	0.79
	Phi sp.t.	0.37	0.18	0.64	0.27	0.04	0.74
	Group Rho	0.06	−0.16	0.36	0.01	−0.23	0.25
	SD sp.	0.12	0.08	0.18	0.18	0.09	0.30
	SD sp.t.	0.11	0.06	0.19	0.15	0.07	0.26
**Breast**	Phi sp.	–	–	–	0.20	0.03	0.67
	Phi sp.t.	–	–	–	0.04	0.00	0.30
	Group Rho	–	–	–	−0.01	−0.29	0.23
	SD sp.	–	–	–	0.06	0.04	0.09
	SD sp.t.	–	–	–	0.10	0.06	0.14
**Cervix**	Phi sp.	–	–	–	0.66	0.17	0.97
	Phi sp.t.	–	–	–	0.21	0.01	0.86
	Group Rho	–	–	–	−0.03	−0.31	0.23
	SD sp.	–	–	–	0.23	0.14	0.36
	SD sp.t.	–	–	–	0.22	0.12	0.38
**Colorectal**	Phi sp.	0.28	0.06	0.70	0.08	0.01	0.49
	Phi sp.t.	0.03	0.00	0.23	0.07	0.01	0.48
	Group Rho	−0.01	−0.27	0.23	0.01	−0.23	0.25
	SD sp.	0.10	0.06	0.14	0.12	0.08	0.17
	SD sp.t.	0.14	0.09	0.20	0.12	0.07	0.19
**Head and neck**	Phi sp.	0.13	0.02	0.48	0.09	0.02	0.39
	Phi sp.t.	0.10	0.01	0.71	0.10	0.02	0.55
	Group Rho	0.01	−0.22	0.26	−0.02	−0.27	0.21
	SD sp.	0.18	0.12	0.25	0.32	0.23	0.47
	SD sp.t.	0.16	0.07	0.31	0.25	0.11	0.44
**Liver**	Phi sp.	0.76	0.23	0.98	0.33	0.10	0.73
	Phi sp.t.	0.14	0.01	0.65	0.27	0.04	0.84
	Group Rho	0.00	−0.25	0.25	0.01	−0.22	0.27
	SD sp.	0.33	0.22	0.49	0.31	0.16	0.64
	SD sp.t.	0.19	0.09	0.40	0.33	0.13	0.75
**Lung**	Phi sp.	0.56	0.21	0.86	0.78	0.42	0.96
	Phi sp.t.	0.34	0.09	0.86	0.27	0.07	0.64
	Group Rho	0.01	−0.25	0.27	0.01	−0.23	0.26
	SD sp.	0.17	0.13	0.22	0.19	0.14	0.27
	SD sp.t.	0.03	0.01	0.07	0.07	0.03	0.14
**Melanoma**	Phi sp.	0.28	0.06	0.77	0.42	0.25	0.63
	Phi sp.t.	0.33	0.05	0.80	0.32	0.14	0.56
	Group Rho	0.00	−0.32	0.32	0.01	−0.22	0.24
	SD sp.	0.15	0.09	0.25	0.18	0.11	0.27
	SD sp.t.	0.11	0.05	0.24	0.19	0.12	0.30
**Pancreas**	Phi sp.	0.27	0.08	0.63	0.10	0.01	0.77
	Phi sp.t.	0.13	0.00	0.79	0.07	0.00	0.62
	Group Rho	0.00	−0.23	0.25	0.00	−0.24	0.24
	SD sp.	0.14	0.07	0.25	0.12	0.05	0.25
	SD sp.t.	0.17	0.09	0.30	0.11	0.02	0.25
**Stomach**	Phi sp.	0.29	0.07	0.69	0.33	0.10	0.69
	Phi sp.t.	0.09	0.01	0.41	0.07	0.01	0.39
	Group Rho	0.01	−0.24	0.27	−0.08	−0.44	0.18
	SD sp.	0.19	0.10	0.35	0.19	0.10	0.36
	SD sp.t.	0.14	0.06	0.34	0.39	0.22	0.65

### Fixed effects

**Fig 2:**
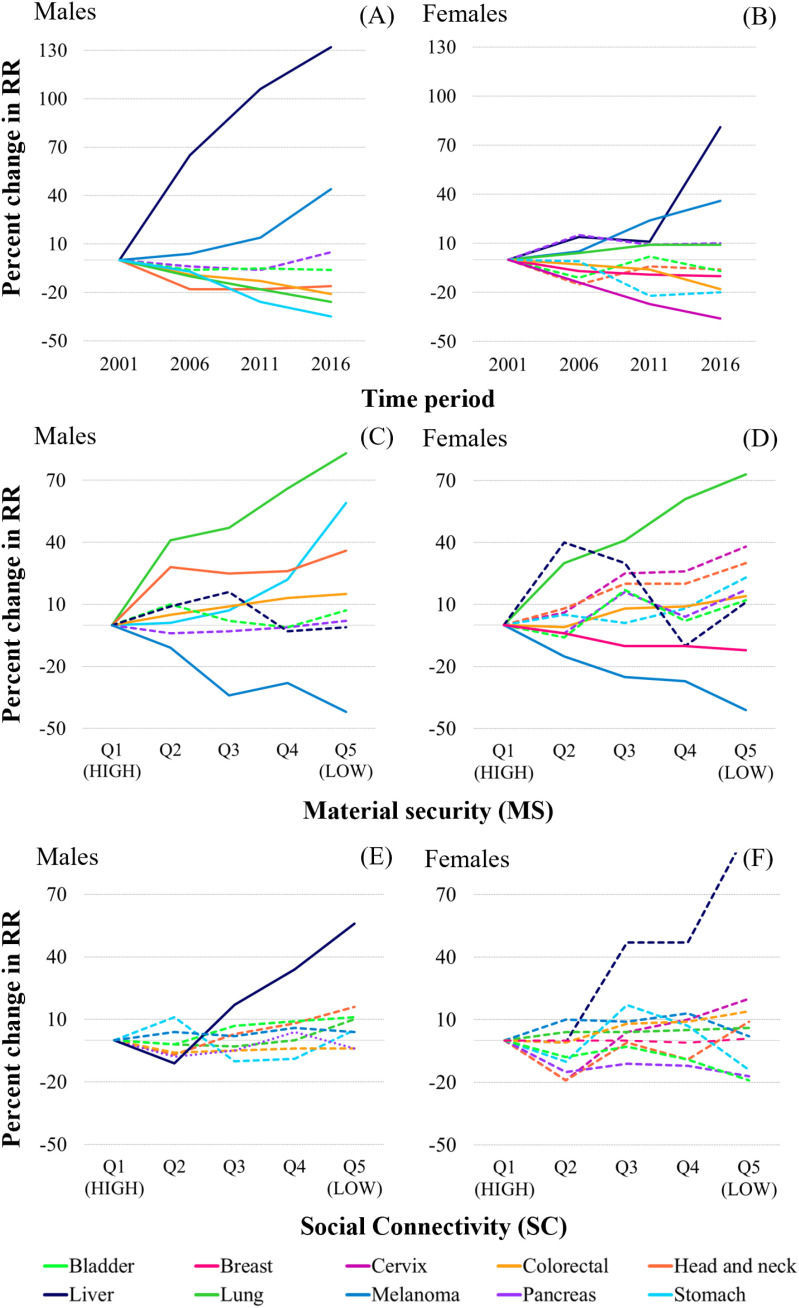
Percent change in male and female cancer-specific posterior median relative risk (RR) in relation to time (A, B), MS (C, D) and SC (E, F). Solid lines indicate statistically significant effects.

Both the direction and effect size of cancer-specific posterior median relative risk (RR) varied considerably over time and in relation to MS and SC (Fig 2A–F, S1 and S2 Tables), however risk did not vary significantly in relation to urbanicity for any of the cancers considered (S3 Table). In males, modeled RRs decreased 35%, 26%, 21%, and 16%, for cancers of the stomach, lung, colorectal and head and neck, respectively, over the period of 2001–2016 (Fig 2A, S1 Table). Those for liver and melanoma increased 132% and 44%, respectively, over the same period (Fig 2A, S1 Table). In females, modeled RRs decreased 36% and 18% for cervical and colorectal, respectively, over the period of 2001–2016 (Fig 2B, S1 Table). RRs for stomach cancer in females also declined over time, although the effect size was about half of that seen in males (−20%) and the downward trend was statistically non-significant. Similar to males, liver and melanoma increased significantly over time in females. In 2016, median RRs for these cancers were 81% and 36% higher than in 2001, respectively. Lung cancer risk in females increased 9% after 2001, a pattern contrasting with the significant decline in lung cancer in males.

Community-level SES (as measured by MS) was an important predictor for five of the eight preventable cancers considered in males, including: lung, stomach, head and neck, colorectal, and melanoma (Fig 2C, S2 Table). Risk increased significantly with decreasing MS, except for melanoma where the risk was lower in communities reporting lower levels of MS. Relative to males from communities with the highest levels of MS (Q1, i.e., referent population), males in communities ranking in the second (Q2), third (Q3), fourth (Q4) and fifth (Q5 – lowest MS), quintiles of the MS gradient, were 41%, 47%, 66% and 83% more likely to develop lung cancer, respectively. Overall increased risk along the gradient ranged from 1% (Q2) and up to 59% (Q5) for stomach; 28% (Q2) and up to 36% (Q5) for head and neck; and 5% (Q2) and up to 15% (Q5) for colorectal cancers. The median RR for melanoma was 42% lower for males in communities reporting the lowest level of MS (Q5).

Similar relationships between cancer risk and community-level MS were observed in females (Fig 2D, S2 Table). For lung cancer, median RRs in females from low MS communities were on average 73% higher than those of females from high MS; those in communities ranking in second (Q2), third (Q3), fourth (Q4) quintiles of the MS gradient had an average excess risk of 30%, 41% and 61%, respectively. Females from low MS communities also had a 14% higher risk of colorectal cancer as compared to those from high MS communities. Non-significant but monotonic increases with decreasing MS, were also seen for cancers of the cervix and head and neck for which the risk was 38% and 30% higher in low MS (Q5) as compared to high MS (Q1) communities, respectively. As for males, median RR for melanoma was 41% lower in females from communities reporting the lowest level of MS (Q5). These females also had a 12% risk reduction for breast cancer as compared to those living in high MS communities.

The associations between SC and cancer risk were generally non-significant, except for male liver cancer (Fig 2E, F, S2 Table). Males from low SC communities (Q5 – lowest SC) were at a 56% increased risk for liver cancer as compared to referent (Q1 – highest SC). Results also showed that females from low SC communities, could be experiencing a doubling in liver cancer risk, although the association was non-significant. Non-significant but elevated RRs were also noted in low SC communities for male head and neck (16%) and lung (10%) cancers as well as for female cervical cancer (20%).

### Spatial random effects

**Fig 3:**
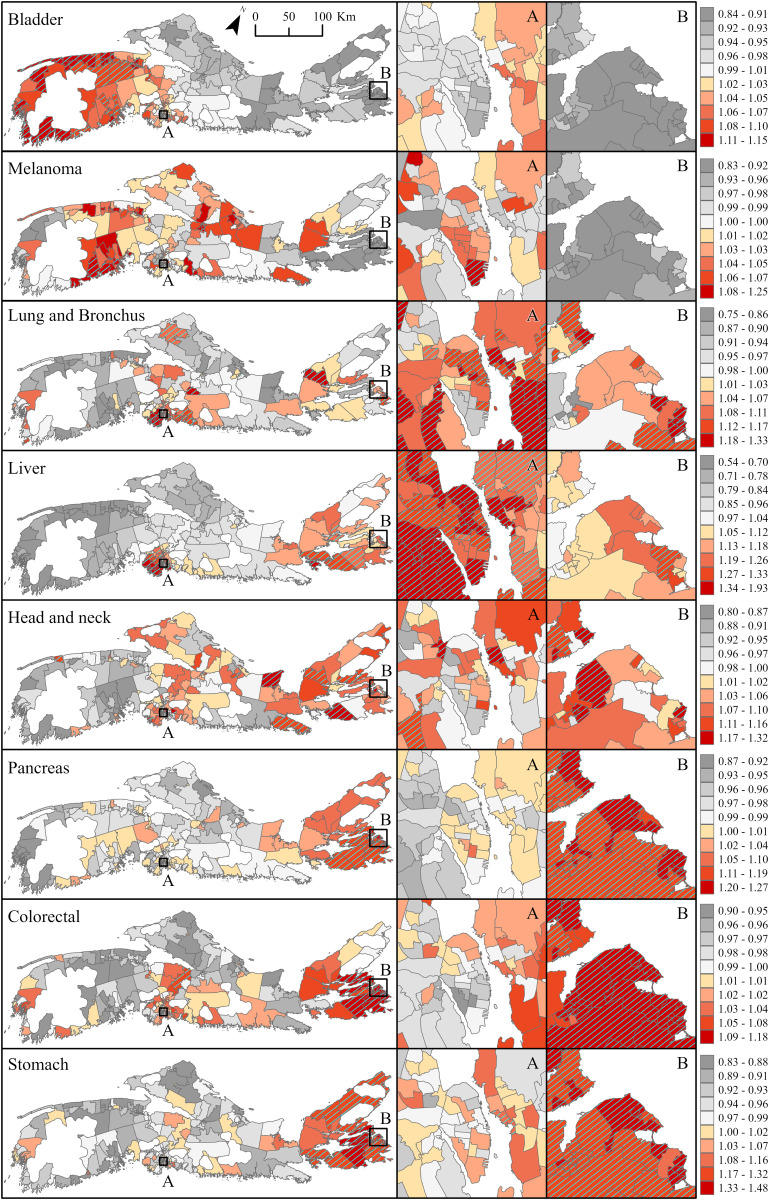
Posterior spatial random effects displaying median relative risk (RR) with overlay of exceedance probability (P_high_≥ 0.8) for eight preventable cancers in males, Nova Scotia 2001-2017. Insets A and B represent the densely populated areas of Halifax and Sydney, respectively. Base Map Source: Statistics Canada, Census Dissemination Areas Boundary File, 17 Nov 2021. Reproduced and distributed on an “as is” basis with the permission of Statistics Canada. [19].

**Fig 4:**
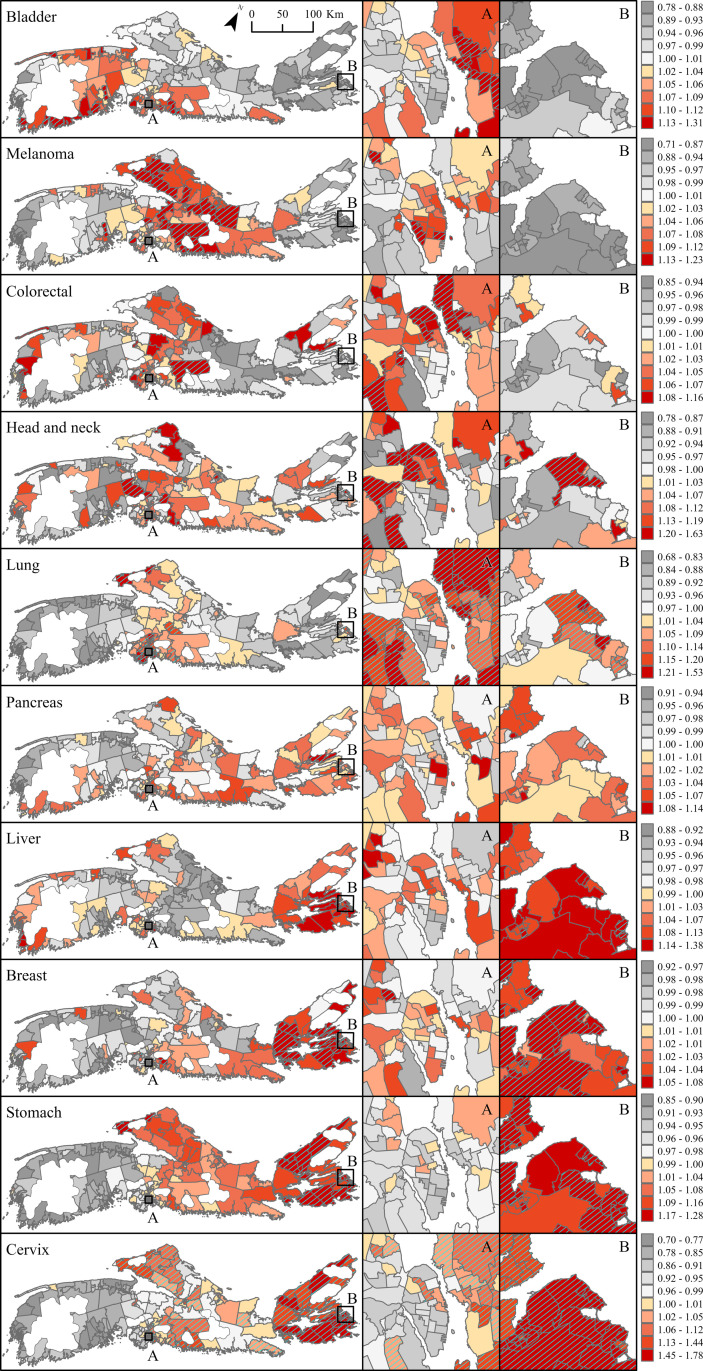
Posterior spatial random effects displaying median relative risk (RR) with overlay of exceedance probability (P_high_≥ 0.8) for ten preventable cancers in females, Nova Scotia 2001-2017. Insets A and B represent the densely populated areas of Halifax and Sydney, respectively. Base Map Source: Statistics Canada, Census Dissemination Areas Boundary File, 17 Nov 2021. Reproduced and distributed on an “as is” basis with the permission of Statistics Canada. [19].

Posterior spatial random effects, representing the persistent spatial variations in median RRs (and 95% credible intervals) over the entire study period, are shown in Fig 3 (males) and Fig 4 (females). These ‘residual’ patterns in cancer risk—independent of measured factors such as time, SES or urbanicity— reveal substantial disparities across NS communities, and a strong west-to-east-gradient. Focusing on communities for which the RRs are > 1 and, considered above average based on an exceedance probability threshold ≥ 80% (P_high_≥ 0.8 – hatched areas on maps), the results show increased risk for male bladder cancer in 51 communities, 45 (88%) of which are located in western NS (Fig 3). Nearly 70% of communities with excess risk for melanoma in males, were also located in this region. In these communities, RRs varied between 1.08 (0.90–1.30) and 1.15 (0.91–1.48) for bladder cancer, and between 1.12 (0.86–1.48) and 1.25 (0.98–1.70) for melanoma. Increased risk for male lung and liver cancers was largely clustered in south-central NS where 48 of the 67 communities with excess lung and 61 of the 68 communities with excess liver cancer, were located (Fig 3). Median RRs in these communities ranged between 1.10 (0.89–1.36) and 1.29 (0.99–1.72) for lung cancer; and between 1.20 (0.78–1.91) and 1.93 (1.17–3.45) for liver cancer. All other communities with increased risk for these cancers were on Cape Breton Island in eastern NS, where the risk ranged between 1.11 (0.88–1.38) and 1.33 (1.06–1.67) for lung cancer; and between 1.25 (0.75–2.10) and 1.42 (0.74–2.87) for liver cancer. Increased risk of pancreatic, colorectal and stomach cancers was also seen in male residing in Cape Breton (Fig 3). Of the 47 communities on the island, 34 had excess pancreatic cancer (RRs between 1.12 (0.86–1.48) and 1.27 (0.98–1.84)); 30 had excess colorectal (RRs between 1.08 (0.91–1.27) and 1.18 (1.01–1.44)); and 42 had excess stomach cancer (RRs between 1.19 (0.79–1.69) and 1.48 (1.03–2.41)). More than half (55%) of all communities in that area had excess risk for all three cancer types. Risk for male head and neck cancer was also elevated in the region, impacting about a fifth of the communities.

Posterior spatial random effects in females followed a largely similar geographic pattern to those observed in males, but with some notable differences (Fig 4). For example, communities with increased risk for female melanoma were largely in northern (14 of 27 communities with P_high_≥ 0.8) and south-central NS (11 of 27 communities with P_high_≥ 0.8). Two were from south-western NS where increased melanoma risk was observed in males. RRs of similar magnitude were observed across all three regions and ranged between 1.12 (0.86–1.54) and 1.23 (0.94–1.71). Communities with increased bladder cancer risk (18 in total with P_high_≥ 0.8) were distributed equally between south-western and south-central NS. However, while communities with excess female bladder cancer coincided largely with those found to have excess risk in males, none of the communities with increased risk in south-central NS, overlapped between sexes. RRs ranged between 1.17 (0.83–1.69) and 1.29 (0.88–1.97) in south-western areas, and between 1.16 (0.83–1.62) and 1.31 (0.94–1.90) in south-central NS. Overall, 82% of communities (69 of 84 communities with P_high_≥ 0.8) with increased lung cancer risk in females, were in south-central NS, a pattern consistent with males (Figs 3 and 4). The median RRs across these communities ranged between 1.10 (0.87–1.37) and 1.53 (1.20–1.99). Only three communities had increased liver cancer risk in females, and one had increased pancreatic cancer; all were located on Cape Breton Island. RRs for liver cancer ranged between 1.30 (0.74–2.80) and 1.38 (0.75–3.34), and that for pancreatic cancer was about half the magnitude (RRs 1.14 (0.88–1.55)). The majority of communities with increased risk for stomach cancer were on Cape Breton Island (23 of 25 communities with P_high_≥ 0.8), and most overlapped with areas where excess risk was also seen in males. Communities with increased risk for breast and cervical cancers also clustered in the region. Of the 22 communities with excess breast cancer, 18 were in Cape Breton and all, also had excess cervical cancer. Overall, 45 of the 47 communities on the island had higher-than average rate of cervical cancer as compared to NS as a whole. The effect size for breast cancer was modest, with RRs ranging between 1.05 (0.94–1.20) and 1.08 (0.97–1.24). For cervix the effect size was larger, RRs ranged between 1.22 (0.78–1.98) and 1.78 (1.16–2.79).

### Posterior predictions

**Fig 5:**
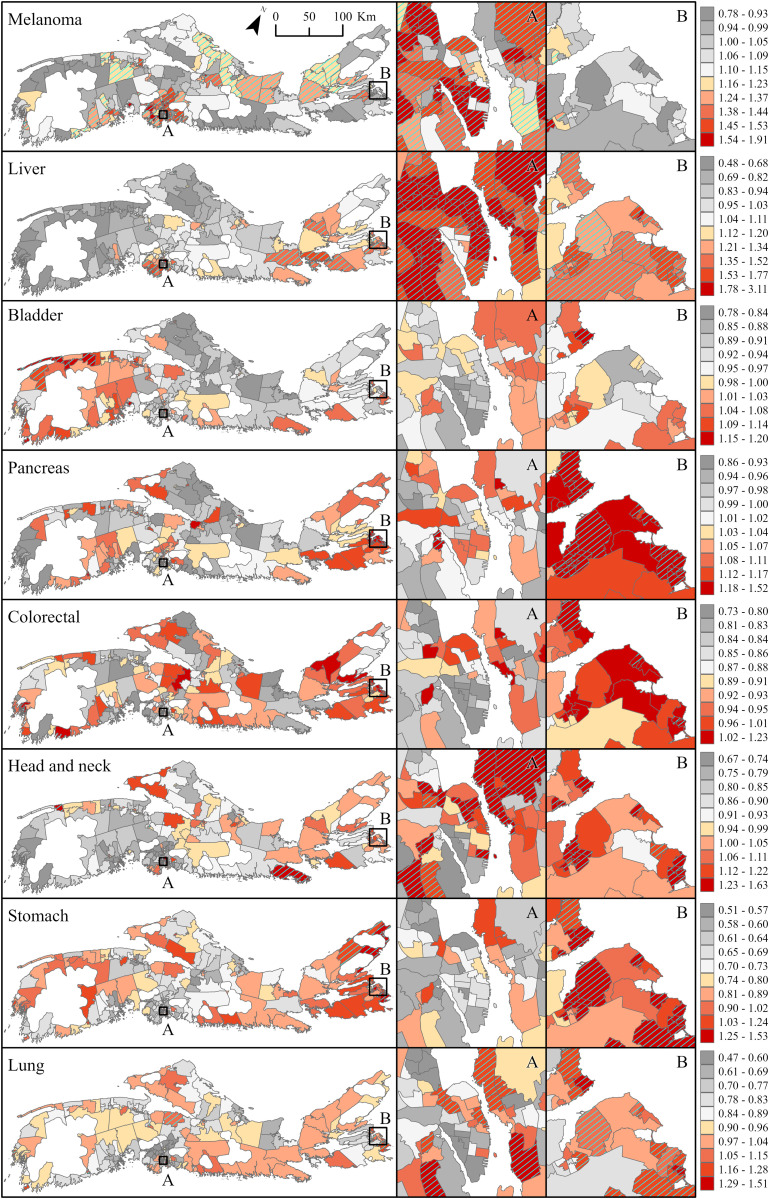
Posterior predictions displaying median relative risk (RR) with overlay of exceedance probability (P_high_≥ 0.8) for eight preventable cancers in males, Nova Scotia 2014-2017. Insets A and B represent densely populated areas of Halifax and Sydney, respectively. Base Map Source: Statistics Canada, Census Dissemination Areas Boundary File, 17 Nov 2021. Reproduced and distributed on an “as is” basis with the permission of Statistics Canada [19].

**Fig 6:**
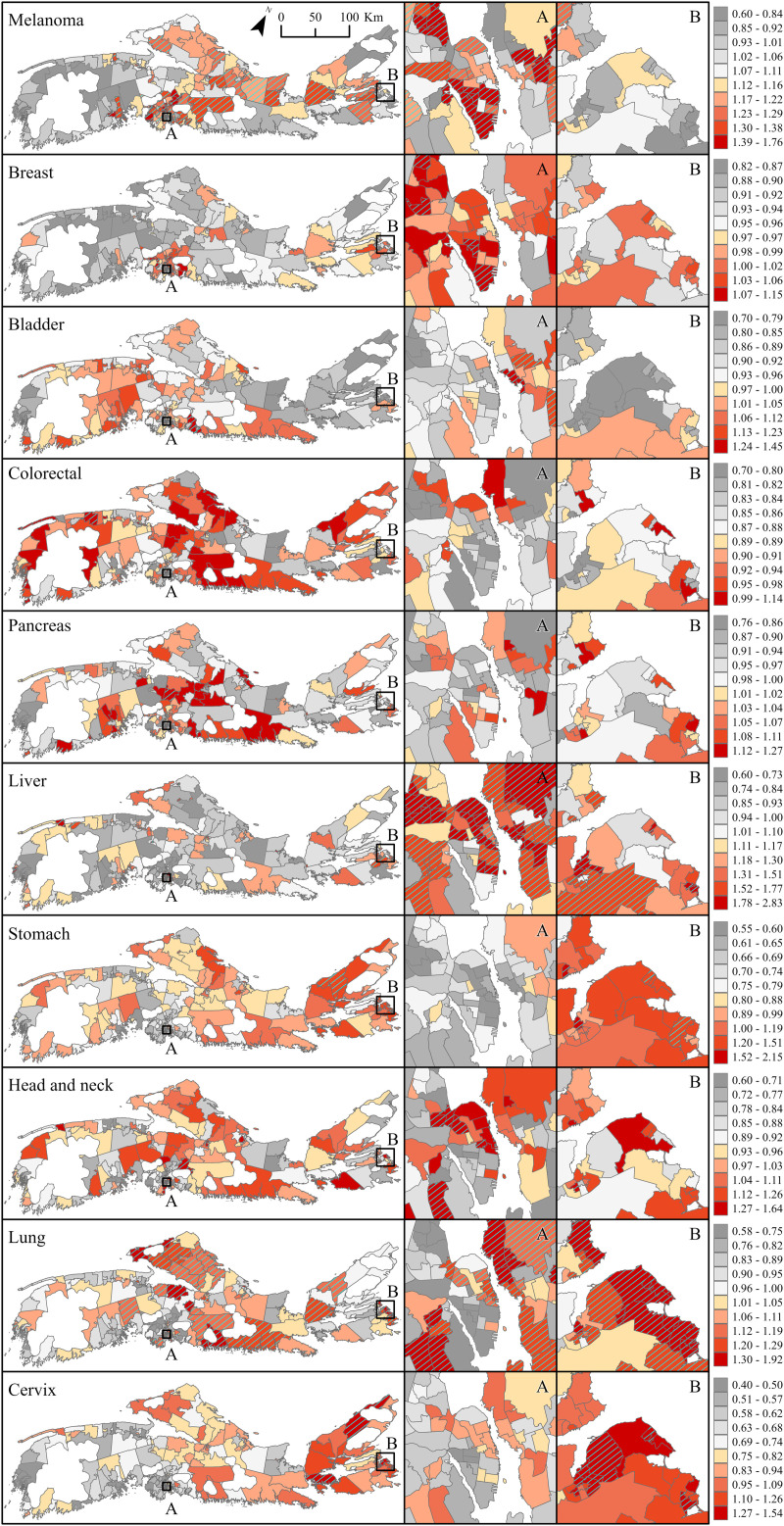
Posterior predictions displaying median relative risk (RR) with overlay of exceedance probability (P_high_≥ 0.8) for ten preventable cancers in females, Nova Scotia 2014-2017. Insets A and B represent densely populated areas of Halifax and Sydney, respectively. Base Map Source: Statistics Canada, Census Dissemination Areas Boundary File, 17 Nov 2021. Reproduced and distributed on an “as is” basis with the permission of Statistics Canada [19].

Posterior predictions representing the median RRs (and 95% credible intervals) in each community over the period centered on 2016 (i.e., data years 2014–2017) relative to that of NS measured over the entire study period (2001–2017), are shown in Figs 5 and 6 for males and females, respectively. Those associated with all four time periods, providing a more complete visualization of how cancer risk changed over time and across space, are presented in supplementary material (S1–S10 Figs). Again, focusing on communities for which the RRs are > 1, and considered above average based on an exceedance probability threshold ≥ 80% (P_high_≥ 0.8 – hatched areas on maps), the results highlight significant excess risk for melanoma in males in almost half of the communities (139 of 301 communities). The largest cluster was in the densely populated Halifax area in south-central NS (89 of 139 communities; Fig 5, S1 FigA). Overall, the median RRs across these communities ranged between 1.61 (0.81–1.65) and 1.91 (1.32–2.89). About 30% (94 of 301) of communities were at an increased risk for liver cancer. These areas were clustered in the Halifax areas (63 of 94 communities with P_high_≥ 0.8), and Sydney areas on Cape Breton Island (29 or 94 communities with P_high_≥ 0.8; Fig 5, S2 FigA). Median RRs across these communities ranged between 1.32 (0.60–2.44) and 3.11 (1.75–6.08). Thirteen of 14 communities with increased male bladder cancer risk were located in the south-west of mainland NS (RRs between 1.13 (0.85–1.5) and 1.2 (0.89–1.62)), and 20 of 21 communities with increased pancreatic cancers were in eastern NS, in Cape Breton County (RRs between 1.22 (0.80–2.0) and 1.52 (0.96–2.41); Fig 5, S3 and S4 FigsA). For period 2016, only three communities had excess male colorectal cancer compared to 96 for period 2001 (Figs 5, S5 FigA). All three were in Cape Breton NS (RRs between 1.14 (0.85–1.55) and 1.23 (0.89–1.74)). Increased risk for lung and head and neck cancers was found in about 10% of communities, and largely clustered around the Halifax and Sydney areas (Fig 5; S6 and S8 FigsA). Median RRs for these cancer types ranged between 1.11 (0.88–1.39) and 1.51 (1.19–1.92) for lung; and between 1.20 (0.79–1.80) and 1.63 (1.09–2.50) for head and neck. For the period centered on 2016, 19 communities had excess stomach cancer in males, all located on Cape Breton Island. Looking back in time, communities with increased risk for male stomach cancer were distributed almost equally between mainland NS and Cape Breton Island (S7 FigA).

Posterior predictions in females followed a similar pattern to those observed in males for melanoma, liver, and stomach cancers, but notable differences were seen for all other cancer types (Fig 6). For melanoma, the results showed significant excess risk (i.e., RRs are > 1, P_high_≥ 0.8) in about one third of the communities (97 of 301 communities). As in males, the largest cluster was around Halifax (68 of 97 communities; Fig 6, S1 FigB). Median RRs were of similar magnitude as those in males, ranging between 1.19 (0.79–1.80) and 1.76 (1.35–2.78). All seven communities with increased bladder cancer risk were in south-central NS, whereas for earlier periods, excess risk was also seen in south-west NS (S3 FigB). Median RRs for this cancer ranged between 1.20 (0.78–1.86) and 1.45 (0.90–2.42). Only two communities had increased risk for pancreatic cancer with effect size of similar magnitude to males. About 5% of communities had excess risk for head and neck cancers with no evidence of spatial clustering. Median RRs ranged between 1.36 (0.66–2.88) and 1.64 (0.76–3.66) for head and neck. As in males, communities with increased risk for liver cancers clustered around Halifax (38 of 61 communities with P_high_≥ 0.8) and Sydney areas (14 of 61 communities with P_high_≥ 0.8); those with increased stomach cancer spanned across Cape Breton Island (6 of 7 communities with P_high_≥ 0.8). Median RRs ranged between 1.47 (0.55–3.66) and 2.83 (1.13–8.52) for liver cancer, and between 1.42 (0.63–3.47) and 2.15 (0.98–5.22) for stomach. Increased risk for lung cancers was observed in about 30% of communities (87 of 301) and these clustered around Halifax and Sydney areas as seen in males, but also in north-central NS (Fig 6; S8 FigB; RRs between 1.11 (0.86–1.43) and 1.92 (1.44–2.56)). Finally, 13 communities had increased risk for breast cancer, and 10 had increased risk for cervical cancer. All communities with excess breast cancer were within the Halifax area; communities with excess cervical cancer were on Cape Breton Island (Fig 6). These findings offer an important contrast to the posterior predictions for the period 2001 which showed 112 communities with excess breast cancer, and 91 with excess cervical cancer (S9 and S10 Figs), distributed more evenly across the province. For period 2001, the RRs ranged between 1.10 (0.88–1.36 and 1.29 (1.03–1.62) for breast; and between 1.29 (0.70–2.30) and 2.90 (1.50–5.90) for cervix; for 2016, the RRs ranged between 1.10 (0.88–1.39) and 1.15 (0.94–1.44) for breast; and between 1.32 (0.67–2.46) and 1.54 (0.77–3.16) for cervix.

**Fig 7:**
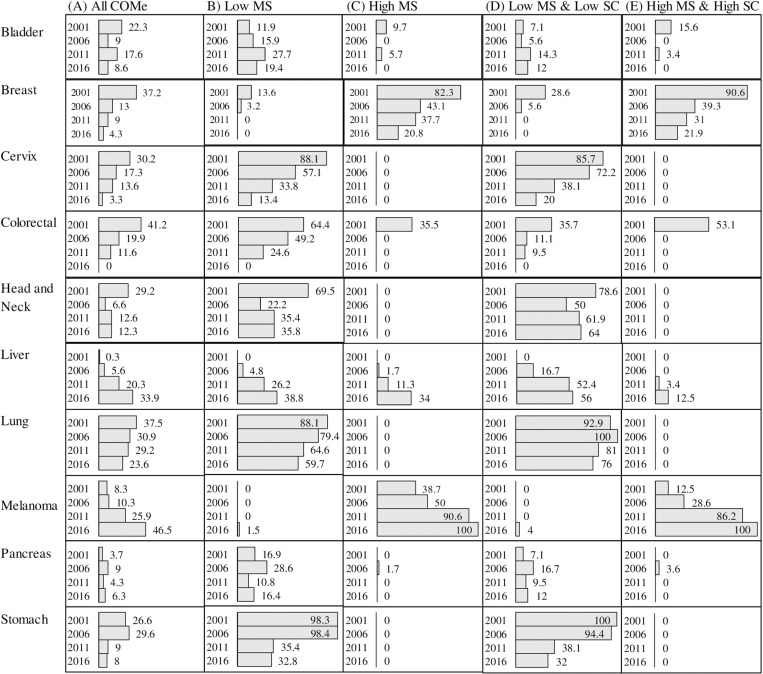
Percentage of communities (COMe) with significant increased cancer risk based on exceedance probabilities (P_high_≥ 0.8) for the posterior predicted median relative risk (RR) estimated for each time period and cancer type, both sexes combined. Results are presented for all COMe (A), communities ranking in either the lowest (B) or highest (C) level of material security (MS) and those reporting both low levels of MS and social connectivity (SC), (D) or both high levels of MS and SC (E).

For both sexes combined, the percentage of communities with excess risk (RRs > 1 and P_high_≥ 0.8) based on posterior predictions, decreased over time for cancers of the breast, cervix, colorectum, lung and stomach; and, increased for liver cancer and melanoma (Fig 7). However, the results also showed that communities with excess cancer risk were disproportionally represented by communities reporting ‘low MS’ (Fig 7B) or combined ‘low MS and low SC’ (Fig 7D). This was consistent across cancer types and time periods, except for breast cancer and melanoma for which high SES communities (i.e., ‘high MS’ (Fig 7C) or combined ‘high MS and high SC’ (Fig 7E)) were most impacted. The results also showed that the unequal distribution in risk across communities with different SES profile has become exacerbated over time. For example, increased risk for cervical cancer was observed in 30% of the 301 NS communities studied in period 2001, but in 86% of those reporting both ‘low MS and low SC’. In period 2016, these figures dropped to 3% and 20%, respectively. As such, low SES communities were being disproportionally represented by a factor of three in 2001 and by about a factor of six in 2016. Communities with excess head and neck, or lung cancers, were being disproportionally represented by communities reporting both ‘low MS and low SC’. For example, in period 2016, 64% of communities with ‘both low MS and low SC’ (Fig 7D) had excess head and neck cancer, in comparison to 36% of low MS (Fig 7B) and 12% of ‘all communities’ (Fig 7A). The pattern was also seen for liver cancer, except that for this cancer type, communities with low or high MS were equally at increased risk. Finally, patterns for colorectal cancer were unique in that more than one third of all communities had excess risk in period 2001 regardless of SES profiles, impacting communities with both ‘high MS and high SC’ almost as much as those with ‘low MS’. In period 2016, it is the only cancer type for which none of the communities have elevated risk, regardless of SES profile. However, one must note that analyses stratified by sex, showed that one community had increased risk of female colorectal cancer and three at increased risk of male, as detailed earlier.

### Similarity in cancer profile

**Fig 8:**
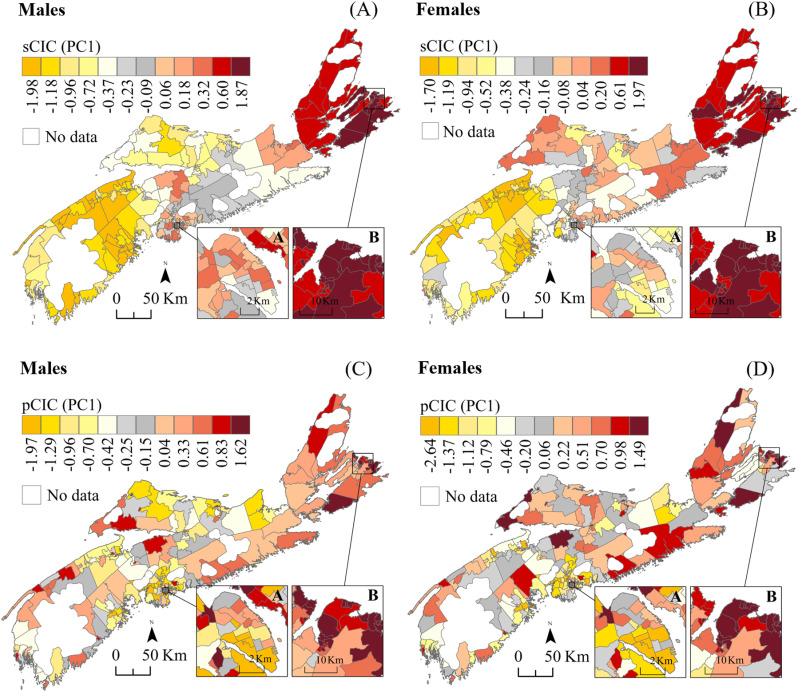
Sex-specific Composite Index of Cancer of spatial random effects (sCIC, A-B) and predictions (pCIC, C-D) based on 8 cancer types in males, and 10 in females. Negative values for sCIC (yellow shades) are associated with higher occurrence of bladder cancer and melanoma in males; and higher occurrence of bladder cancer in females. Positive values for sCIC (orange-red shades) are associated with higher occurrence of stomach, colorectal and pancreatic cancers in males; and higher occurrence of cervical, stomach and breast cancers in females. Negative values for pCIC (yellow shades) are associated with higher occurrence of melanoma in both males and females. Positive values for pCIC (orange-red shades) are associated with higher occurrence of lung and stomach in males; and higher occurrence of cervical and lung cancers in females. Insets (A) and (B) represent the densely populated areas of Halifax and Sydney, respectively. Base Map Source: Statistics Canada, Census Dissemination Areas Boundary File, 17 Nov 2021. Reproduced and distributed on an “as is” basis with the permission of Statistics Canada [19].

Fig 8 shows sex-specific scores for the ‘spatial’ and ‘predicted’ Composite Index of Cancer (sCIC and pCIC), summarizing the correlation structure between community-level cancer-specific median RRs for the spatial random effects and posterior predictions. The results highlight patterns of similarity or dissimilarity in cancer profiles across communities, with communities ranking at opposite ends of the gradient of scores being impacted by different types of cancers. The component loadings associated with the sCIC and pCIC help identify the dominant cancers driving these regional differences in risk profile (Table 3). Focusing on sCIC scores, which reflect persistent patterns of joint spatial distribution of cancers as observed over the entire study period, the results showed a strong spatial gradient in cancer profile, along a west-to-east axis consistent across sexes (Pearson correlation 0.90, p<0.0001). Based on sCIC loadings, the results suggest that males in communities from western NS have been at an increased risk of bladder cancer and melanoma over the 16-year study period; and those in communities from eastern NS, have been disproportionally impacted by joint excess of stomach, colorectal and pancreatic cancers (Fig 8A; Table 3). Some similarities in males’ cancer profile can also be seen between communities from south-central and eastern NS, a pattern likely associated with joint increased risk for lung and liver cancers. Over the years, communities from western NS have also been at increased risk of female bladder, whereas those in eastern NS have had excess co-occurrence of cervical, stomach and breast cancers amongst females. Overall, the sCIC scores accounted for 50.6% and 38.1% of the total variance associated with spatial random effects in males and females, respectively (Table 3).

**Fig 9:**
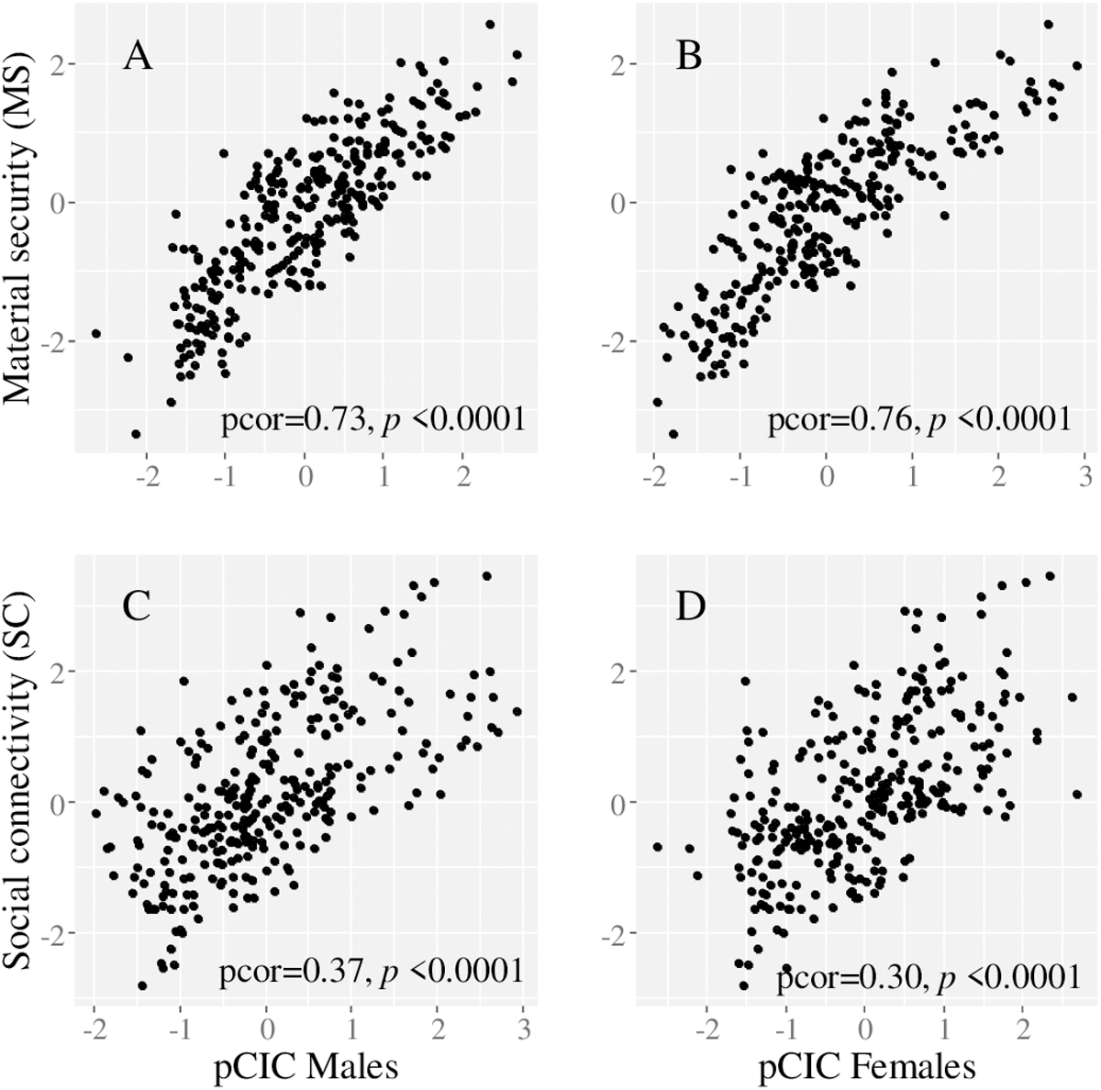
Scatterplot of the Composite Index of Cancer for posterior predictions (pCIC) and community socioeconomic status as measured with material security (A, B) or social connectivity (C, D), in males and females. Pearson partial correlations (pcor) and associated p-values are included to show the strength and significance of the associations.

Focusing on pCIC scores, reflecting patterns of joint spatial distribution of cancers for the most recent period (2016: 2014–2017 data), the results showed important spatial heterogeneity in cancer profiles (Fig 8C, D). For males, the pCIC loadings (Table 3) suggested that communities with highest pCIC scores had joint excess risk for stomach and lung cancers, and those ranking at the opposite and lower end of the pCIC gradient of scores, had increased risk for melanoma. Comparative figures for females pointed to joint excess risk for cervical and lung cancers in communities with highest pCIC scores; and excess melanoma in communities with lowest pCIC, as seen in males. For both sexes, the results also showed contrasting cancer profiles between north and south-end communities of the Halifax peninsula area (Fig 8C, D, A-insets). Overall, the pCIC scores accounted for 45.4% and 32.9% of the total variance associated with median posterior predicted RRs in males and females, respectively (Table 3). For both sexes, the scores were strongly correlated with community-level SES (Fig 9), and in particular with material security (Fig 9A, B).

**Table 3:** Loadings of the Composite Index of Cancer for posterior predictions (pCIC 2014-2017) and spatial random effects (sCIC 2001-2017), for 10 preventable cancers in males and females, Nova Scotia.

Males				Females			
Posterior predictions		Spatial random effects		Posterior predictions		Spatial random effects	
Cancer type	pCIC loadings	Cancer type	sCIC loadings	Cancer type	pCIC loadings	Cancer type	sCIC loadings
Melanoma	−0.70	Bladder	−0.71	Melanoma	−0.71	Bladder	−0.68
Liver	0.30	Melanoma	−0.70	Breast	−0.39	Melanoma	−0.43
Bladder	0.46	Lung	0.44	Bladder	−0.22	Colorectal	−0.18
Pancreas	0.56	Liver	0.60	Colorectal	0.21	Head and neck	0.19
Colorectal	0.75	Head and neck	0.65	Pancreas	0.24	Lung	0.19
Head and neck	0.77	Pancreas	0.81	Liver	0.51	Pancreas	0.55
Stomach	0.82	Colorectal	0.82	Stomach	0.62	Liver	0.72
Lung	0.84	Stomach	0.87	Head and neck	0.69	Breast	0.80
				Lung	0.80	Stomach	0.87
				Cervix	0.85	Cervix	0.92
Eigenvalues:	3.6		4.0	Eigenvalues:	3.3		3.8
% variance:	45.4		50.6	% variance:	32.9		38.1

## Discussion

This study observed important variations in cancer risk mediated by space, time, and the SES conditions experienced by communities. For non-sex specific cancers, these associations were typically more pronounced in males. The decreasing trends in the RR for cancers of the stomach, colorectum, head and neck, breast, cervix and lung amongst males observed in this study, were consistent with trends reported for Canada in general [36–38] and other high-income countries such as the US [39–42] and UK [43–45]. Much of this downward trajectory in cancer rates has been attributed to a changing landscape in smoking and cancer screening, as well as vaccination [39,46–48]. Similarly, the upward trends in liver cancer and melanoma have also been reported for Canada as a whole and elsewhere [38,49–54]. Migrant populations from countries with high prevalence of hepatitis B virus (HBV) have been reported to contribute to the increasing trend of liver cancer in the US, Australia and Canada [53,55,56]. Obesity, high alcohol consumption, diabetes, non-alcoholic fatty liver disease, and possibly a change in cancer coding and reporting practices, are amongst other factors potentially contributing to the increasing trend in liver cancer [50]. Variations in the stability of the ozone layer as well as tanning behaviour combined with inadequate sun protective measures have been associated with the increasing trend in melanoma [52,57].

This study also identified a nuanced relationship between cancer risk and community-level SES. High MS communities were found to exhibit higher risks of melanoma and breast cancers; while low MS communities were at an increased risk of lung, stomach, head and neck, colorectal and cervical cancers. The findings were largely consistent with the literature [51,58–72] and have been attributed to the differential prevalence of cancer-causing factors in the various population segments of the SES gradient. For example, behavioural cancer risk factors such as smoking, alcohol use, physical inactivity, obesity, and unbalanced diet are more prevalent in materially disadvantaged communities and reflect the social determinants of health [59,60,62,73–82]. Tope et al [72], also suggest that the higher prevalence of infectious agents (i.e., *Helicobacter pylori*, HBV and HBC) amongst individuals of lower household income might be contributing to increased risk for cancers of the stomach and liver in Canada. In addition, the generally lower adherence to cancer screening guidelines amongst individual of lower household income could further contribute to excess risk for colorectal and cervical cancers [72,83]. The relationship between cancer screening behaviour and SES is, however, complex. This is because screening for colorectal and cervical cancer aims to detect precancerous conditions, whereas screening for other cancers such as breast, aims to identify pre-existing cancers at an earlier stage [72]. Therefore, while poor adherence to screening guidelines amongst materially disadvantaged communities may result in increased incidence of colorectal and cervical cancers, higher screening rates amongst high MS communities could contribute to higher incidence of breast cancer amongst this population segment. Differences in reproductive behaviours between individuals in low and high MS communities further contribute to these nuanced relationships, whereby lower parity amongst wealthier individuals may be protective against cervical cancer but could result in increased breast cancer risk [72,84]. Finally, several explanations have been proposed for the increased incidence in melanoma among individuals of higher SES, including greater access to and awareness of screening, intermittent intense sun exposure through more frequent vacations in sunny climates, and increased leisure time outdoors [36,51,72,85,86].

In this study, MS was a stronger predictor of cancer risk than SC, except for liver cancer for which increased risk was observed in low SC communities. The influence of social deprivation, as opposed to material deprivation, on cancer risk is not well understood. However, loneliness and social isolation have been associated with increased cancer mortality, lower survival, and more recently, increased total cancer incidence—independent of behavioural factors such as alcohol consumption, physical activity and smoking [87–90]. Other studies have also demonstrated that the interplay between behavioural/psychosocial processes and immune factors could support a favourable environment for cancer development and progression [91–94]. In addition, the joint occurrence of many of the prevailing risk factors in low or high SES communities can work together to exacerbate the health consequences through possible synergistic effects. For example, while high SES groups may consume comparable or greater amounts of alcohol than people from disadvantaged neighbourhoods, the latter are more likely to consume alcohol as part of a suite of health challenging behaviours, resulting in greater alcohol-attributable harm [22,95,96]. Therefore, these individual and area-level mediating risk factors can operate distinctly or intersect to compound their effect on health.

Overall, the work presented here highlighted significant inequities in cancer risk operating beyond the control of an individual. Further, the examination of risk variation in relation to SES over time, revealed that inequities in risk distribution were not only sustained over the entire study period, but also worsened—a pattern observed for eight of the ten preventable cancers studied. The comparatively higher percentage of low-SES communities with excess risk in recent time, suggests that much of the reduction in risk has been concentrated in the more privileged segment of the NS population. The trend was particularly striking for cancers of the head and neck, lung and cervix – three cancers known to be between 85% and almost 100% preventable [97]. These findings are consistent with the growing SES-related health inequalities previously reported in Atlantic Canada and other Canadian provinces [98]. They also align with the temporal evolution of income polarization seen in Canada where over a 40-year period, the low-income population group has become proportionally larger with the continued erosion of the middle-class [99]. The resulting widening gap between the most and least socioeconomically secure population segments has been suggested to be an important determinant of health linked to poorer health outcomes [100–104]. Moving beyond a ‘single cancer’ to a multivariate analysis combining cancer-specific posterior predictions from ‘multiple cancer types’ (the composite indicator of cancer analysis) also confirmed that the primary gradient in cancer risk profile was significantly correlated with community-level SES. The results suggest that SES not only influences the risk of developing a certain type of cancer, but also the risk of being diagnosed with geographically co-occurring cancers. The contrasting cancer profile observed on the Halifax peninsula, an area less than 20 km^2^, that would otherwise be considered homogenous, further highlighted the value of using small-areas analysis to inform and strengthen cancer prevention efforts. The SES profile of Halifax north- and south-end communities has been historically different, and this study shows that this differential distribution of material and social wealth matters to our health [17,105].

The intrinsic link between geography and cancer was demonstrated throughout the study. Spatial processes were significant for all cancer types, with neighbourhood effects being especially pronounced in males for cancers of the liver, bladder, lung, and, in females for lung and cervical cancers. The multivariate analysis combining cancer-specific spatial random effects, confirmed the presence of a strong west-to-east underlying gradient in cancer risk profile driven by large-scale geographic processes. This spatial structure was unexpected and underscores the importance of persistent and unmeasured factors; processes that could operate over extended periods, including environmental, occupational, or potentially gene by environment interactions. For example, bladder and melanoma were two co-occurring cancers contributing to the residual cancer risk observed in communities from southwest mainland NS. Both cancers have been linked to environmental and occupational risk factors [85,106–110]. Bedrock formations in the region are an important source of geogenic arsenic in well water [111–114], a known environmental carcinogen that has been associated with many cancers, including bladder and melanoma [110,113,115]. In NS, increased risk of bladder cancer has been associated with exposure to arsenic levels below current recommended regulatory limit [116]. In addition, the latitude of the southwest portion of NS is amongst the southernmost part of Canada and may result in greater everyday exposure to higher ambient summer UVR which has been associated with increased melanoma [85,117].

Communities ranking at the other extreme of the spatial gradient and located in eastern NS offered a contrasting cancer profile that highlighted the joint occurrence of stomach, pancreatic, colorectal in males and the co-occurrence of stomach, cervical, and breast cancers in females. All these cancers have been associated with a broad range of risk factors related to behaviour, occupation or genetics [97,118–130]. The respective and possibly interactive effects on these co-occurring cancers have not been measured in this study and cannot be assessed. However, as the SES indicators included in our models served as proxies to key behavioural risk factors, the observed residual excess risk may be associated with occupational or genetic factors. Of the five co-occurring cancers, three—cancers of the breast, colorectum and pancreas—were shown to have stronger links to inherited genetic factors [130,131]. However, as the contribution of hereditary factors to the development of cancer is known to be relatively minor [130–132], other drivers may also be at play. For instance, occupational exposure to crystalline silica has been associated with increased stomach cancer amongst mining and foundry workers [133]. Stomach along with many other cancers including, pancreatic, rectal, breast and possibly cervical cancers—have also been associated with the metalworking fluids industry [134–136]. Shift work which affects hormonal balance and disrupts circadian rhythms has been associated with increased breast cancer risk [137,138]. Whether these types of occupational exposures have contributed to the joint cancer profile in eastern NS, remains to be evaluated.

This study has many important strengths. First, the work includes cancer incidence data from a population-based cancer registry that follows national and international registration standards. This adherence ensures consistent data collection and completeness over time, improving the utility and statistical power of trend analysis. Second, the systematic collection of residential civic address at time of diagnosis by cancer registrars, allowed for almost 90% of the incident cases to be georeferenced to a community with high degree of certainty, reducing location misclassification. Third, the statistical method employed to estimate cancer risk accounted for spatial and spatio-temporal dependencies. This lowers the likelihood of Type I error, which consists of identifying an area as having higher-than-average risk when its true rate is, in fact, comparable to background level (i.e., sex-specific NS average over entire study period). Similarly, the application of a local conditional autoregressive model for spatially autocorrelated errors, and lag 1 autoregressive model for temporally related observations to the correlation matrices of the MS and SC indices, ensured a more robust estimation of the area-based SES. Nonetheless, this study has some limitations. First, residential history is not captured in the NS Cancer Registry and as such it was not possible to account for patterns of mobility. The civic address of patients at time of diagnosis, while being highly precise, may not accurately reflect past exposures because some individuals may have moved. Inevitably, this could have resulted in some exposure misclassification and in our ability to account for exposures to latent environmentally and socially driven processes. It may also have caused the SES indicators to incompletely capture the excess risk associated with SES-related behavioural factors, especially in those communities that have experienced long-lasting low-SES living conditions, many of which are located in eastern NS. Unaccounted patterns of mobility are often considered a weakness to the spatial analysis of diseases with long latency such as cancer. However, the influence of misclassification due to mobility is more likely to result in more homogeneity in spatial patterning of cancer. The inclusion of spatial and spatio-temporal random effects helped absorb this source of variability, allowing the detection of spatial clusters. Of course, clusters may reflect past exposures that continue to prevail in the impacted communities or exposures that are no longer present. Clusters can also result from a non-random displacement of socially and materially disadvantage population groups—who may have had a history of exposures to cancer-causing factors—to areas of greater affordability. Either way, this study is not designed to establish causality but rather, to identify communities with increased cancer risk and possibly in need of added support to adopt health promoting behaviours, and to manage the impact of a cancer diagnosis. Second, about 10% of the incident cases included in the study were georeferenced using residential postal codes known to have varying positional accuracy [139] which could have resulted in location misclassification and bias, especially in rural settings. However, an examination of the assigned locations based upon postal codes showed a random distribution of the cases across the province, suggesting error that is not localized to one area, minimizing the risk of spatial bias. Further, a comparison of crude standardised incidence ratios (SIRs) based on ‘all cases’ *versus* those based on ‘cases with high spatial accuracy’, highlighted a strong overall correlation (Pearson correlation = 0.925, P < 0.001), irrespective of sex, time or cancer types (data available upon request).

## Conclusion

Cancer does not develop in isolation from the contextual setting of a community. Cancer prevention is closely linked to the physical, social, cultural and economic environments in which people live. Many interacting exposure environments, operating at various spatial scales and over time, contribute to an individual, community, and population risk of developing cancer, and hence our capacity to prevent cancer. This study provided strong evidence in support of adopting a geographically-focused approach to inform and strengthened cancer prevention efforts. It demonstrated the importance and feasibility of estimating cancer risk over small areas, even within a sparsely populated setting such as that of Nova Scotia. The analysis highlighted notable differences in the cancers impacting communities. It also revealed significant health inequities rooted in the differential distribution of material and social wealth, that have worsened over time. It is well known that people from socially disadvantaged communities tend to experience cancer disproportionally, whether through increased risk of developing the disease, as highlighted in this study, or as other studies have shown through increased risk of dying from cancer. Actions to break this cycle, which reinforces marginalization and inequities at all points of the cancer continuum and over time, are needed. Being able to profile cancer risk locally in small areas, and in relation to a community-level SES, is an important first step to achieving targeted and equitable cancer prevention. The analysis adopted in the NS-Matrix study can be used to identify priority populations for specific cancer prevention interventions—i.e., identifying those communities with higher-than-average risk but with lower capacity to manage the impact of cancer or adopt health promoting behaviours. Thus, the analysis can be used to focus resources where they are most needed. In addition, as exposure to a single or multiple risk factors can result in the development of one or many different types of cancer, having the ability to quantify the range of cancers that co-occur in specific communities also offers an opportunity to target limited resources in a relevant and equitable manner. These insights enable an evidence-based approach that not only enhances our understanding of cancer risk and supports hypothesis generation, but also aids in the equitable targeting and managing of prevention measures. Future studies integrating longitudinal estimates of key cancer-causing factors will further help to elucidate socio-spatial patterns in cancer disparities. To our knowledge, this is the first comprehensive study that utilizes a holistic and yet locally relevant framework to provide baseline data to support effective and equitable cancer prevention. The approach presented here is implementable to other regions and countries to inform and strengthen prevention efforts aiming at reducing the burden of cancer or that of other chronic diseases.

## Supporting information

S1 Table Posterior estimates of fixed effects over time. Posterior estimates of fixed effects (median relative risk (RR)) estimated over time and associated credible intervals (CrIs) for 10 preventable cancers in males, females, and for combined sexes, Nova Scotia 2001–2017. Values in bold indicate statistically significant effects based on 95% CrIs. Base Map Source: Statistics Canada, Census Dissemination Areas Boundary File, 17 Nov 2021. Reproduced and distributed on an “as is” basis with the permission of Statistics Canada [19].
(DOCX)

S2 Table Posterior estimates of fixed effects by SES levels. Posterior estimates of fixed effects (median relative risk (RR)) estimated by levels of material security (MS) and social connectivity (SC) and associated credible intervals (CrIs) for 10 preventable cancers in males, females, and for combined sexes, Nova Scotia 2001–2017. Values in bold indicate statistically significant effects based on 95% CrIs. Base Map Source: Statistics Canada, Census Dissemination Areas Boundary File, 17 Nov 2021. Reproduced and distributed on an “as is” basis with the permission of Statistics Canada [19].
(DOCX)

S3 Table Posterior estimates of fixed effects by urbanicity. Posterior estimates of fixed effects (median relative risk (RR)) estimated by urbanicity status and associated credible intervals (CrIs) for 10 preventable cancers in males, females, and for combined sexes, Nova Scotia 2001–2017. Values in bold indicate statistically significant effects based on 95% CrIs. Base Map Source: Statistics Canada, Census Dissemination Areas Boundary File, 17 Nov 2021. Reproduced and distributed on an “as is” basis with the permission of Statistics Canada [19].
(DOCX)

S1 Fig Posterior predictions for melanoma of the skin by time period. Posterior predictions displaying median relative risk (RR) with overlay of exceedance probability (P_high_≥ 0.8) for melanoma of the skin by time period for males (A) and females (B), Nova Scotia. Insets A and B represent the densely populated areas of Halifax and Sydney, respectively. Base Map Source: Statistics Canada, Census Dissemination Areas Boundary File, 17 Nov 2021. Reproduced and distributed on an “as is” basis with the permission of Statistics Canada [19].
(TIF)

S2 Fig Posterior predictions for liver cancer by time period. Posterior predictions displaying median relative risk (RR) with overlay of exceedance probability (P_high_≥ 0.8) for liver cancer by time period for males (A) and females (B), Nova Scotia. Insets A and B represent the densely populated areas of Halifax and Sydney, respectively. Base Map Source: Statistics Canada, Census Dissemination Areas Boundary File, 17 Nov 2021. Reproduced and distributed on an “as is” basis with the permission of Statistics Canada [19].
(TIF)

S3 Fig Posterior predictions for bladder cancer by time period. Posterior predictions displaying median relative risk (RR) with overlay of exceedance probability (P_high_≥ 0.8) for bladder cancer by time period for males (A) and females (B), Nova Scotia. Insets A and B represent the densely populated areas of Halifax and Sydney, respectively. Base Map Source: Statistics Canada, Census Dissemination Areas Boundary File, 17 Nov 2021. Reproduced and distributed on an “as is” basis with the permission of Statistics Canada [19].
(TIF)

S4 Fig Posterior predictions for pancreatic cancer by time period. Posterior predictions displaying median relative risk (RR) with overlay of exceedance probability (P_high_ ≥ 0.8) for pancreatic cancer by time period for males (A) and females (B), Nova Scotia. Insets A and B represent the densely populated areas of Halifax and Sydney, respectively. Base Map Source: Statistics Canada, Census Dissemination Areas Boundary File, 17 Nov 2021. Reproduced and distributed on an “as is” basis with the permission of Statistics Canada [19].
(TIF)

S5 Fig Posterior predictions for colorectal cancer by time period. Posterior predictions displaying median relative risk (RR) with overlay of exceedance probability (P_high_ ≥ 0.8) for colorectal cancer by time period for males (A) and females (B), Nova Scotia. Insets A and B represent the densely populated areas of Halifax and Sydney, respectively. Base Map Source: Statistics Canada, Census Dissemination Areas Boundary File, 17 Nov 2021. Reproduced and distributed on an “as is” basis with the permission of Statistics Canada [19].
(TIF)

S6 Fig Posterior predictions for head and neck cancer by time period. Posterior predictions displaying median relative risk (RR) with overlay of exceedance probability (P_high_ ≥ 0.8) for head and neck cancer by time period for males (A) and females (B), Nova Scotia. Insets A and B represent the highest population density areas of Halifax and Sydney, respectively. Base Map Source: Statistics Canada, Census Dissemination Areas Boundary File, 17 Nov 2021. Reproduced and distributed on an “as is” basis with the permission of Statistics Canada [19].
(TIF)

S7 Fig Posterior predictions for stomach cancer by time period. Posterior predictions displaying median relative risk (RR) with overlay of exceedance probability (P_high_ ≥ 0.8) for stomach cancer by time period for males (A) and females (B), Nova Scotia. Insets A and B represent the densely populated areas of Halifax and Sydney, respectively. Base Map Source: Statistics Canada, Census Dissemination Areas Boundary File, 17 Nov 2021. Reproduced and distributed on an “as is” basis with the permission of Statistics Canada [19].
(TIF)

S8 Fig Posterior predictions for lung cancer by time period. Posterior predictions displaying median relative risk (RR) with overlay of exceedance probability (P_high_ ≥ 0.8) for lung cancer by time period for males (A) and females (B), Nova Scotia. Insets A and B represent the densely populated areas of Halifax and Sydney, respectively. Base Map Source: Statistics Canada, Census Dissemination Areas Boundary File, 17 Nov 2021. Reproduced and distributed on an “as is” basis with the permission of Statistics Canada [19].
(TIF)

S9 Fig Posterior predictions for breast cancer by time period. Posterior predictions displaying median relative risk (RR) with overlay of exceedance probability (P_high_ ≥ 0.8) for female breast cancer by time period, Nova Scotia. Insets A and B represent the densely populated areas of Halifax and Sydney, respectively. Base Map Source: Statistics Canada, Census Dissemination Areas Boundary File, 17 Nov 2021. Reproduced and distributed on an “as is” basis with the permission of Statistics Canada [19].
(TIF)

S10 Fig Posterior predictions for cervical cancer by time period. Posterior predictions displaying median relative risk (RR) with overlay of exceedance probability (P_high_ ≥ 0.8) for female cervical cancer by time period, Nova Scotia. Insets A and B represent the densely populated areas of Halifax and Sydney, respectively. Base Map Source: Statistics Canada, Census Dissemination Areas Boundary File, 17 Nov 2021. Reproduced and distributed on an “as is” basis with the permission of Statistics Canada [19].
(TIF)
