# Supplementary material for: Small-area spatio-temporal analysis of cancer risk to support effective and equitable cancer prevention
Source: PLoS One. 2025 Jun 9;20(6):e0325523. doi: 10.1371/journal.pone.0325523 (PMC12148161; doi:10.1371/journal.pone.0325523)
Supplement: S1 Table — Posterior estimates of fixed effects (median relative risk (RR)) estimated over time and associated credible intervals (CrIs) for 10 preventable cancers in males, females, and for combined sexes, Nova Scotia 2001–2017. Values in bold indicate statistically significant effects based on 95% CrIs. Base Map Source: Statistics Canada, Census Dissemination Areas Boundary File, 17 Nov 2021. Reproduced and distributed on an “as is” basis with the permission of Statistics Canada [19]. (DOCX) [file pone.0325523.s001.docx]

| **Cancer Type** |  | **Parameter** |  | **Males** | | |  | **Females** | | |  | **Combined Sex** | | |
| --- | --- | --- | --- | --- | --- | --- | --- | --- | --- | --- | --- | --- | --- | --- |
|  |  |  |  | Median | 2.5% | 97.5% |  | Median | 2.5% | 97.5% |  | Median | 2.5% | 97.5% |
|  |  |  |  |  |  |  |  |  |  |  |  |  |  |  |
| **Bladder** |  | 2001 (ref) |  | - | - | - |  | - | - | - |  | - | - | - |
|  |  | 2006 |  | 0.94 | 0.84 | 1.05 |  | 0.89 | 0.74 | 1.07 |  | 0.93 | 0.85 | 1.02 |
|  |  | 2011 |  | 0.95 | 0.85 | 1.06 |  | 1.02 | 0.86 | 1.22 |  | 0.9s8 | 0.90 | 1.08 |
|  |  | 2016 |  | 0.94 | 0.84 | 1.04 |  | 0.93 | 0.78 | 1.12 |  | 0.96 | 0.88 | 1.06 |
| **Breast** |  | 2001 (ref) |  | - | - | - |  |  |  |  |  |  |  |  |
|  |  | 2006 |  | - | - | - |  | **0.93** | **0.88** | **0.98** |  |  |  |  |
|  |  | 2011 |  | - | - | - |  | **0.91** | **0.87** | **0.97** |  |  |  |  |
|  |  | 2016 |  | - | - | - |  | **0.90** | **0.85** | **0.95** |  |  |  |  |
| **Cervix** |  | 2001 (ref) |  | - | - | - |  | - | - | - |  | - | - | - |
|  |  | 2006 |  | - | - | - |  | 0.86 | 0.70 | 1.05 |  | - | - | - |
|  |  | 2011 |  | - | - | - |  | **0.73** | **0.59** | **0.90** |  | - | - | - |
|  |  | 2016 |  | - | - | - |  | **0.64** | **0.51** | **0.80** |  | - | - | - |
| **Colorectal** |  | 2001 (ref) |  | - | - | - |  | - | - | - |  | - | - | - |
|  |  | 2006 |  | **0.91** | **0.85** | **0.99** |  | 0.97 | 0.90 | 1.05 |  | **0.94** | **0.89** | **0.99** |
|  |  | 2011 |  | **0.87** | **0.81** | **0.94** |  | 0.94 | 0.87 | 1.01 |  | **0.90** | **0.85** | **0.95** |
|  |  | 2016 |  | **0.79** | **0.73** | **0.86** |  | **0.82** | **0.75** | **0.89** |  | **0.81** | **0.76** | **0.85** |
| **Head and neck** |  | 2001 (ref) |  | - | - | - |  | - | - | - |  | - | - | - |
|  |  | 2006 |  | **0.82** | **0.72** | **0.94** |  | 0.85 | 0.68 | 1.07 |  | **0.83** | **0.74** | **0.93** |
|  |  | 2011 |  | **0.82** | **0.72** | **0.93** |  | 0.96 | 0.77 | 1.19 |  | **0.86** | **0.77** | **0.96** |
|  |  | 2016 |  | **0.84** | **0.73** | **0.95** |  | 0.94 | 0.75 | 1.17 |  | **0.87** | **0.77** | **0.97** |
| **Liver** |  | 2001 (ref) |  | - | - | - |  | - | - | - |  | - | - | - |
|  |  | 2006 |  | **1.65** | **1.17** | **2.32** |  | 1.14 | 0.66 | 1.96 |  | **1.48** | **1.11** | **1.98** |
|  |  | 2011 |  | **2.06** | **1.48** | **2.88** |  | 1.11 | 0.65 | 1.90 |  | **1.77** | **1.34** | **2.35** |
|  |  | 2016 |  | **2.32** | **1.66** | **3.23** |  | **1.81** | **1.08** | **3.03** |  | **2.16** | **1.64** | **2.85** |
| **Lung** |  | 2001 (ref) |  | - | - | - |  | - | - | - |  | - | - | - |
|  |  | 2006 |  | **0.90** | **0.84** | **0.96** |  | 1.04 | 0.97 | 1.13 |  | 0.96 | 0.91 | 1.01 |
|  |  | 2011 |  | **0.82** | **0.76** | **0.87** |  | **1.09** | **1.01** | **1.17** |  | 0.93 | 0.89 | 0.98 |
|  |  | 2016 |  | **0.74** | **0.69** | **0.79** |  | **1.09** | **1.01** | **1.18** |  | 0.89 | 0.85 | 0.94 |
| **Melanoma** |  | 2001 (ref) |  | - | - | - |  | - | - | - |  | - | - | - |
|  |  | 2006 |  | 1.04 | 0.90 | 1.20 |  | 1.05 | 0.90 | 1.22 |  | 1.05 | 0.94 | 1.17 |
|  |  | 2011 |  | 1.14 | 0.99 | 1.32 |  | **1.24** | **1.07** | **1.43** |  | **1.19** | **1.07** | **1.32** |
|  |  | 2016 |  | **1.44** | **1.25** | **1.66** |  | **1.36** | **1.18** | **1.58** |  | **1.42** | **1.28** | **1.58** |
| **Pancreas** |  | 2001 (ref) |  | - | - | - |  | - | - | - |  | - | - | - |
|  |  | 2006 |  | 0.96 | 0.80 | 1.16 |  | 1.15 | 0.95 | 1.39 |  | 1.05 | 0.92 | 1.20 |
|  |  | 2011 |  | 0.94 | 0.78 | 1.12 |  | 1.09 | 0.90 | 1.31 |  | 1.01 | 0.88 | 1.15 |
|  |  | 2016 |  | 1.05 | 0.87 | 1.26 |  | 1.10 | 0.91 | 1.34 |  | 1.08 | 0.94 | 1.23 |
| **Stomach** |  | 2001 (ref) |  | - | - | - |  | - | - | - |  | - | - | - |
|  |  | 2006 |  | 0.93 | 0.78 | 1.12 |  | 0.99 | 0.76 | 1.29 |  | 0.95 | 0.82 | 1.10 |
|  |  | 2011 |  | **0.74** | **0.61** | **0.89** |  | 0.78 | 0.59 | 1.02 |  | **0.76** | **0.65** | **0.88** |
|  |  | 2016 |  | **0.65** | **0.53** | **0.78** |  | 0.80 | 0.60 | 1.05 |  | **0.70** | **0.60** | **0.82** |

**S1 Table.**  **Posterior estimates of fixed effects over time**. Posterior estimates of fixed effects (median relative risk (RR)) estimated over time and associated credible intervals (CrIs) for 10 preventable cancers in males, females, and for combined sexes, Nova Scotia 2001-2017. Values in bold indicate statistically significant effects based on 95% CrIs.
