# Supplementary figures and images for: Small-area spatio-temporal analysis of cancer risk to support effective and equitable cancer prevention
Source: PLoS One. 2025 Jun 9;20(6):e0325523. doi: 10.1371/journal.pone.0325523 (PMC12148161; doi:10.1371/journal.pone.0325523)

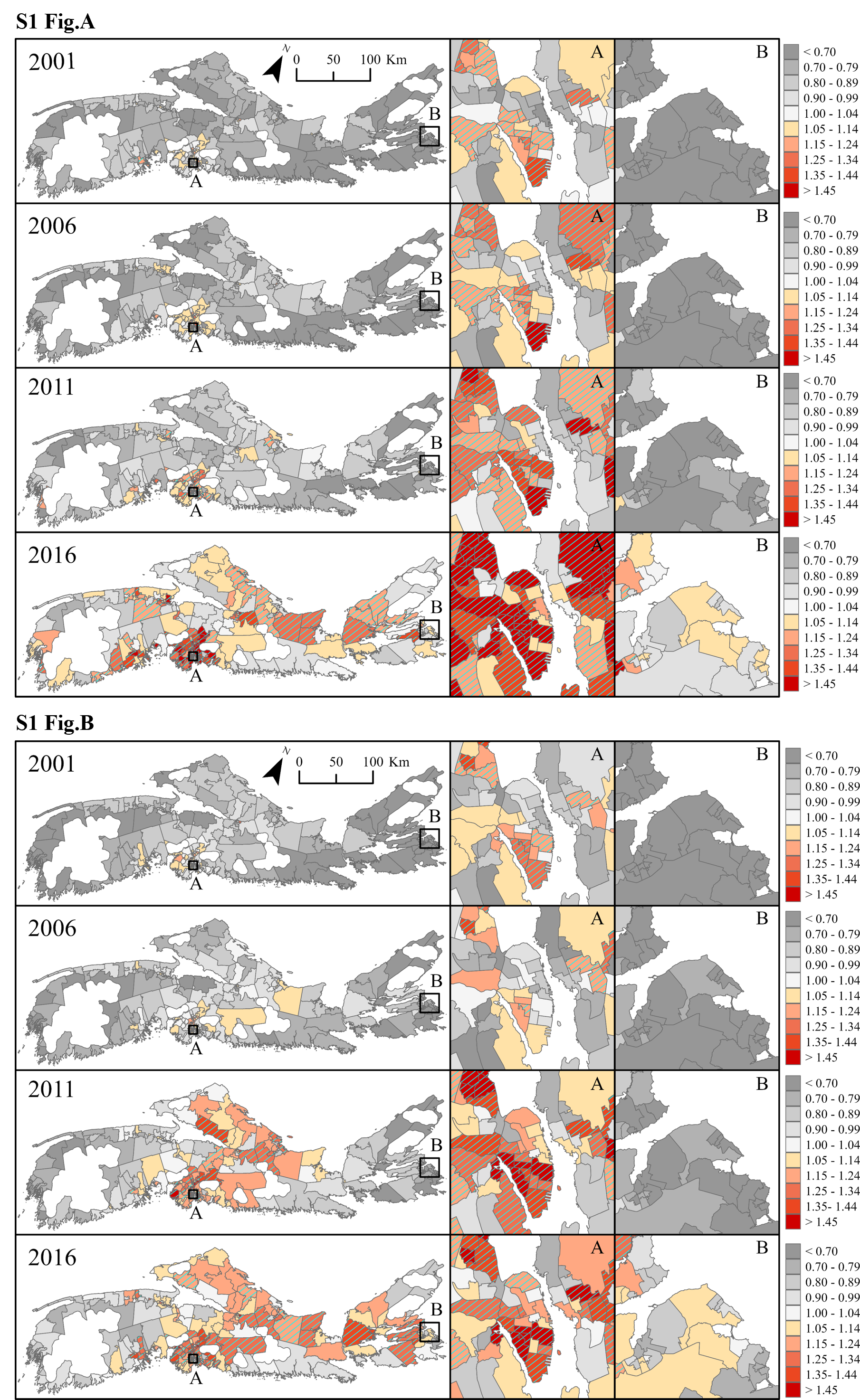

Supplement: S1 Fig — Posterior predictions displaying median relative risk (RR) with overlay of exceedance probability (Phigh≥ 0.8) for melanoma of the skin by time period for males (A) and females (B), Nova Scotia. Insets A and B represent the densely populated areas of Halifax and Sydney, respectively. Base Map Source: Statistics Canada, Census Dissemination Areas Boundary File, 17 Nov 2021. Reproduced and distributed on an “as is” basis with the permission of Statistics Canada [19]. (TIF) [file pone.0325523.s004.tif]

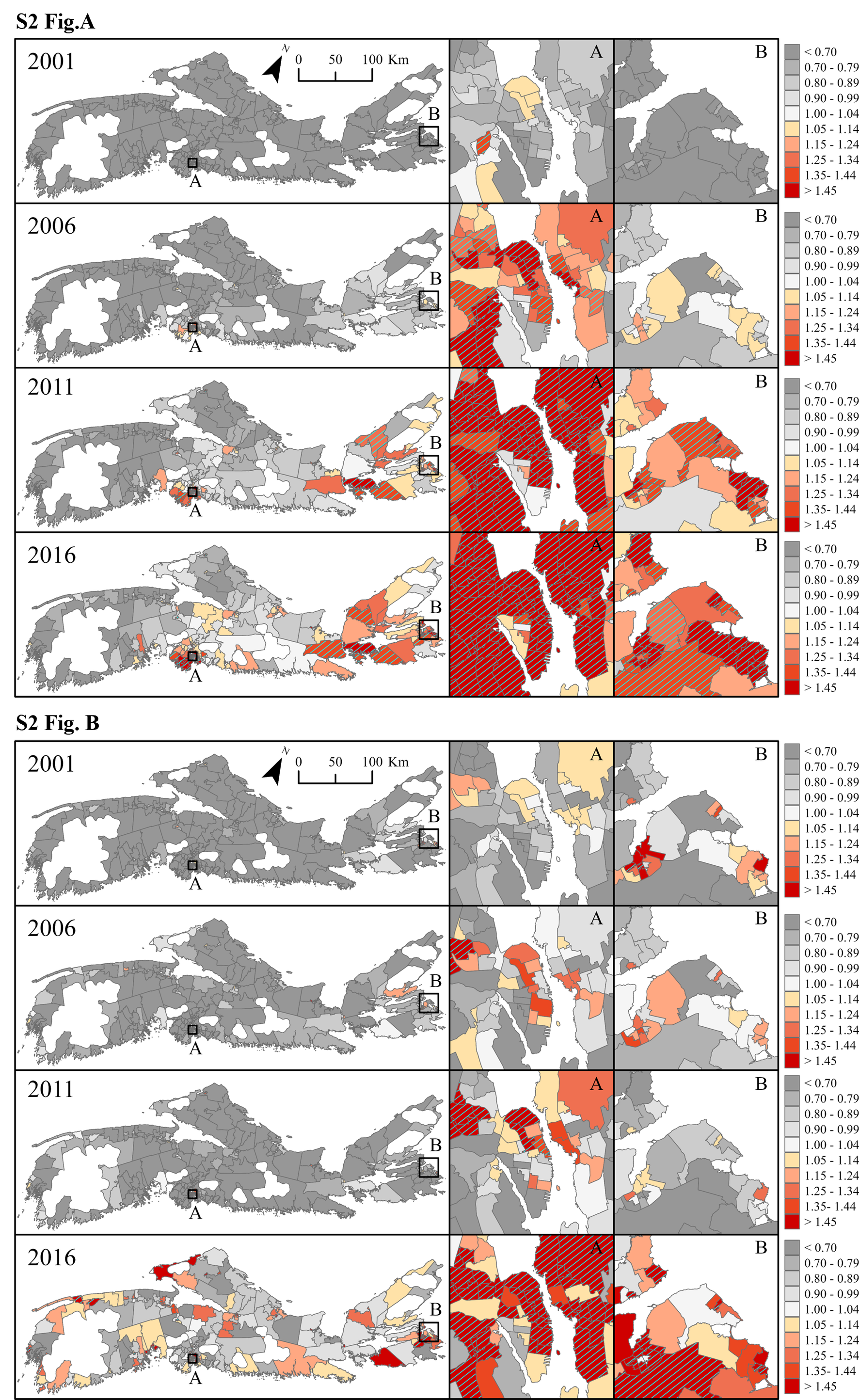

Supplement: S2 Fig — Posterior predictions displaying median relative risk (RR) with overlay of exceedance probability (Phigh≥ 0.8) for liver cancer by time period for males (A) and females (B), Nova Scotia. Insets A and B represent the densely populated areas of Halifax and Sydney, respectively. Base Map Source: Statistics Canada, Census Dissemination Areas Boundary File, 17 Nov 2021. Reproduced and distributed on an “as is” basis with the permission of Statistics Canada [19]. (TIF) [file pone.0325523.s005.tif]

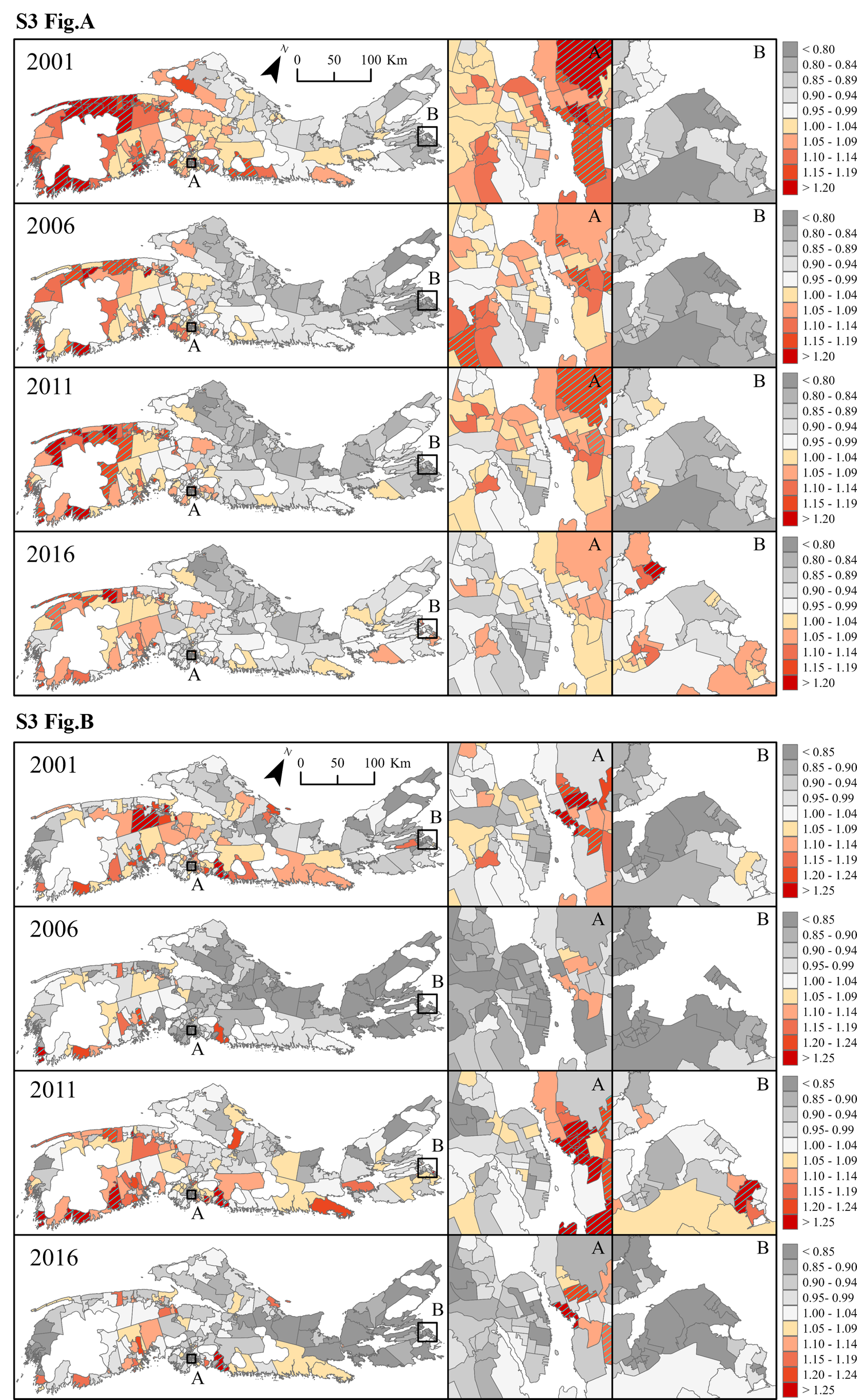

Supplement: S3 Fig — Posterior predictions displaying median relative risk (RR) with overlay of exceedance probability (Phigh≥ 0.8) for bladder cancer by time period for males (A) and females (B), Nova Scotia. Insets A and B represent the densely populated areas of Halifax and Sydney, respectively. Base Map Source: Statistics Canada, Census Dissemination Areas Boundary File, 17 Nov 2021. Reproduced and distributed on an “as is” basis with the permission of Statistics Canada [19]. (TIF) [file pone.0325523.s006.tif]

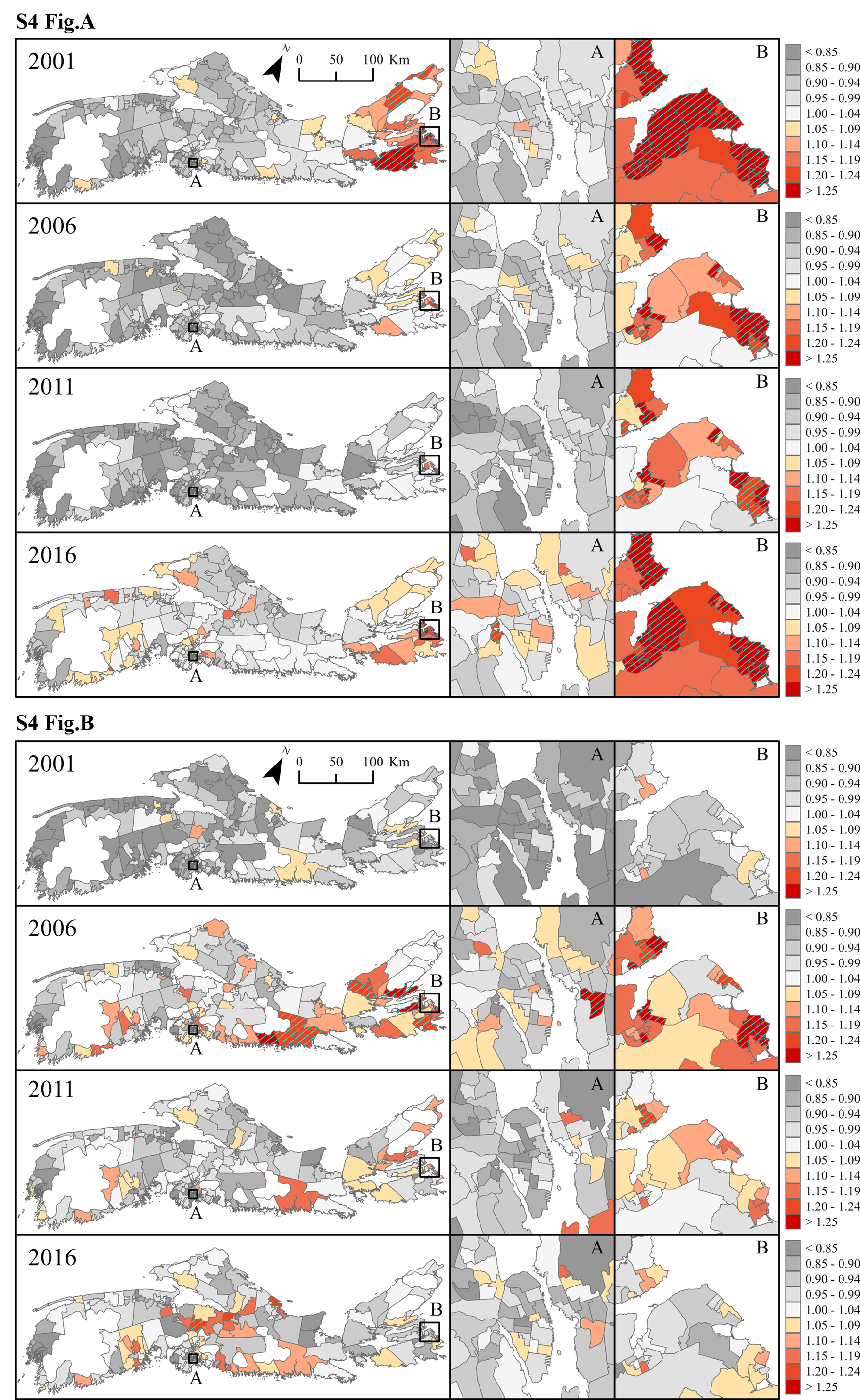

Supplement: S4 Fig — Posterior predictions displaying median relative risk (RR) with overlay of exceedance probability (Phigh ≥ 0.8) for pancreatic cancer by time period for males (A) and females (B), Nova Scotia. Insets A and B represent the densely populated areas of Halifax and Sydney, respectively. Base Map Source: Statistics Canada, Census Dissemination Areas Boundary File, 17 Nov 2021. Reproduced and distributed on an “as is” basis with the permission of Statistics Canada [19]. (TIF) [file pone.0325523.s007.tif]

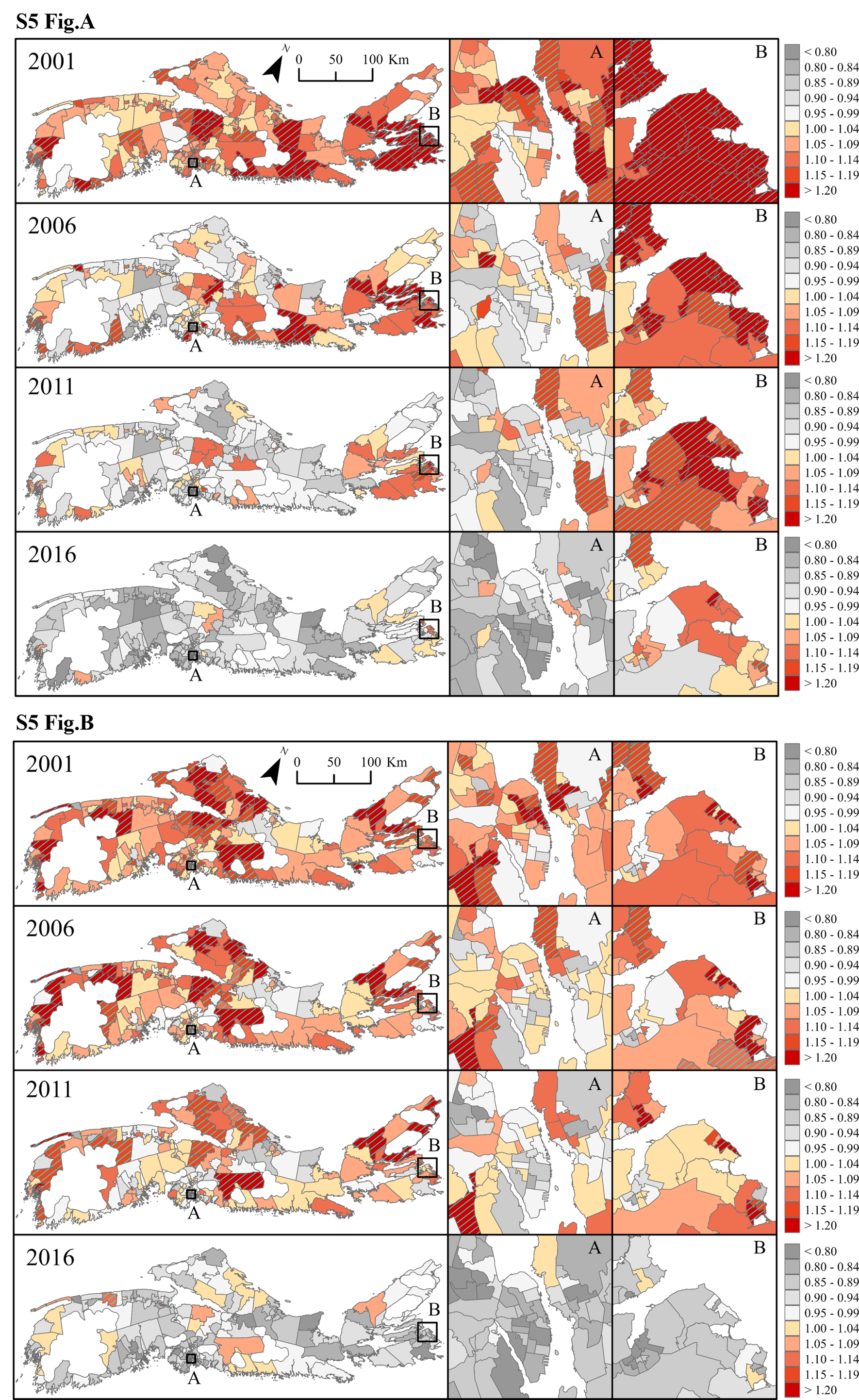

Supplement: S5 Fig — Posterior predictions displaying median relative risk (RR) with overlay of exceedance probability (Phigh ≥ 0.8) for colorectal cancer by time period for males (A) and females (B), Nova Scotia. Insets A and B represent the densely populated areas of Halifax and Sydney, respectively. Base Map Source: Statistics Canada, Census Dissemination Areas Boundary File, 17 Nov 2021. Reproduced and distributed on an “as is” basis with the permission of Statistics Canada [19]. (TIF) [file pone.0325523.s008.tif]

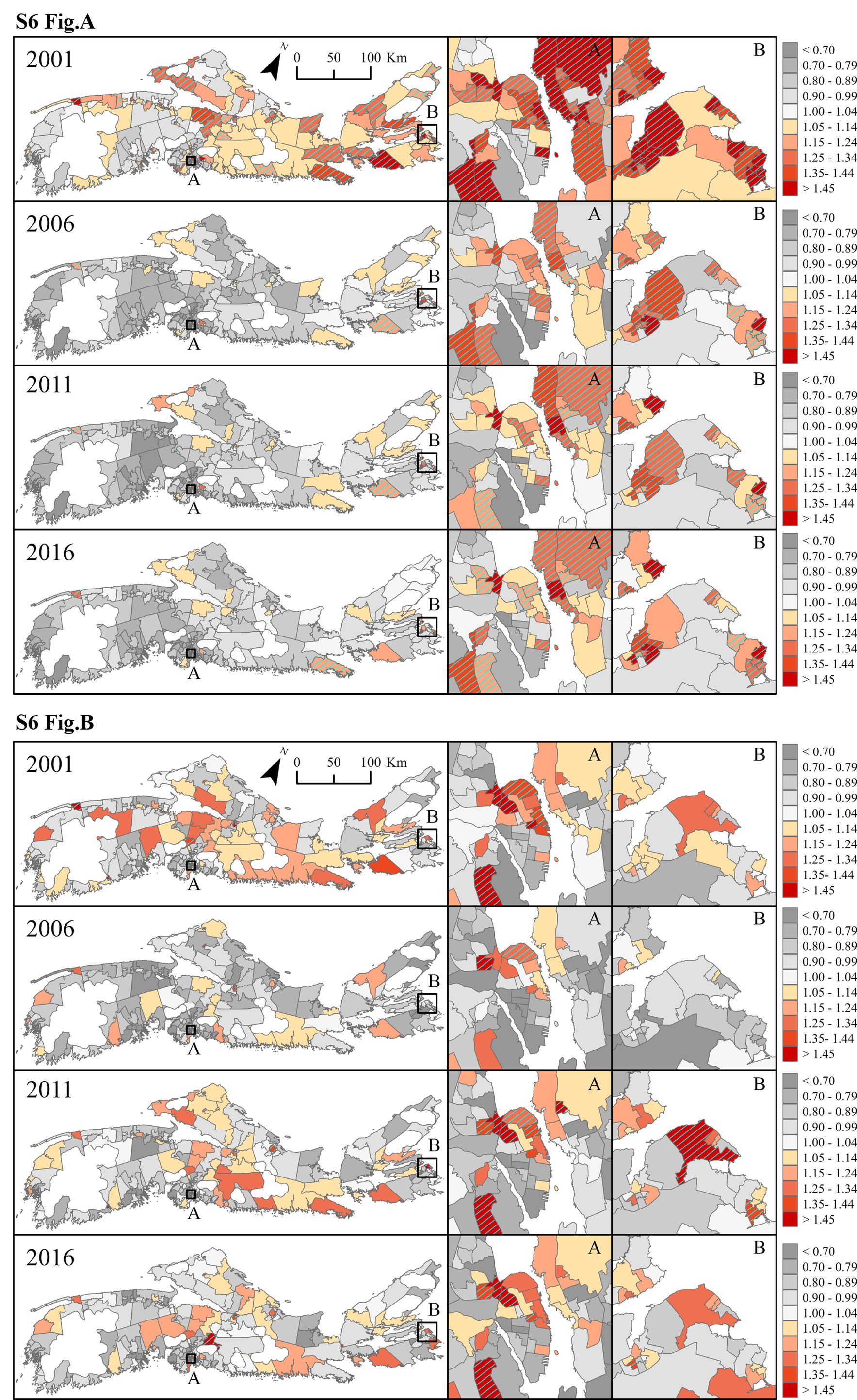

Supplement: S6 Fig — Posterior predictions displaying median relative risk (RR) with overlay of exceedance probability (Phigh ≥ 0.8) for head and neck cancer by time period for males (A) and females (B), Nova Scotia. Insets A and B represent the highest population density areas of Halifax and Sydney, respectively. Base Map Source: Statistics Canada, Census Dissemination Areas Boundary File, 17 Nov 2021. Reproduced and distributed on an “as is” basis with the permission of Statistics Canada [19]. (TIF) [file pone.0325523.s009.tif]

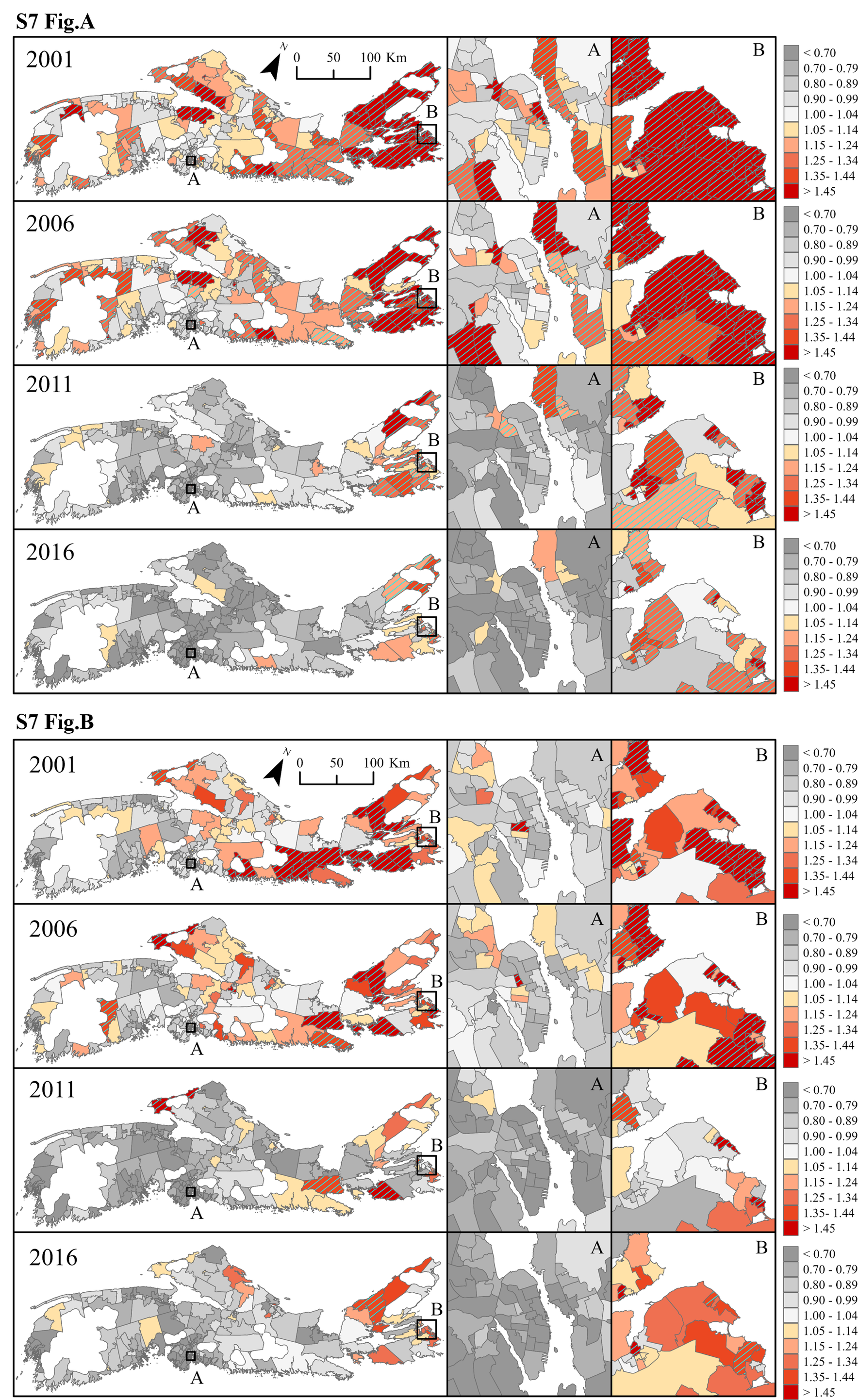

Supplement: S7 Fig — Posterior predictions displaying median relative risk (RR) with overlay of exceedance probability (Phigh ≥ 0.8) for stomach cancer by time period for males (A) and females (B), Nova Scotia. Insets A and B represent the densely populated areas of Halifax and Sydney, respectively. Base Map Source: Statistics Canada, Census Dissemination Areas Boundary File, 17 Nov 2021. Reproduced and distributed on an “as is” basis with the permission of Statistics Canada [19]. (TIF) [file pone.0325523.s010.tif]

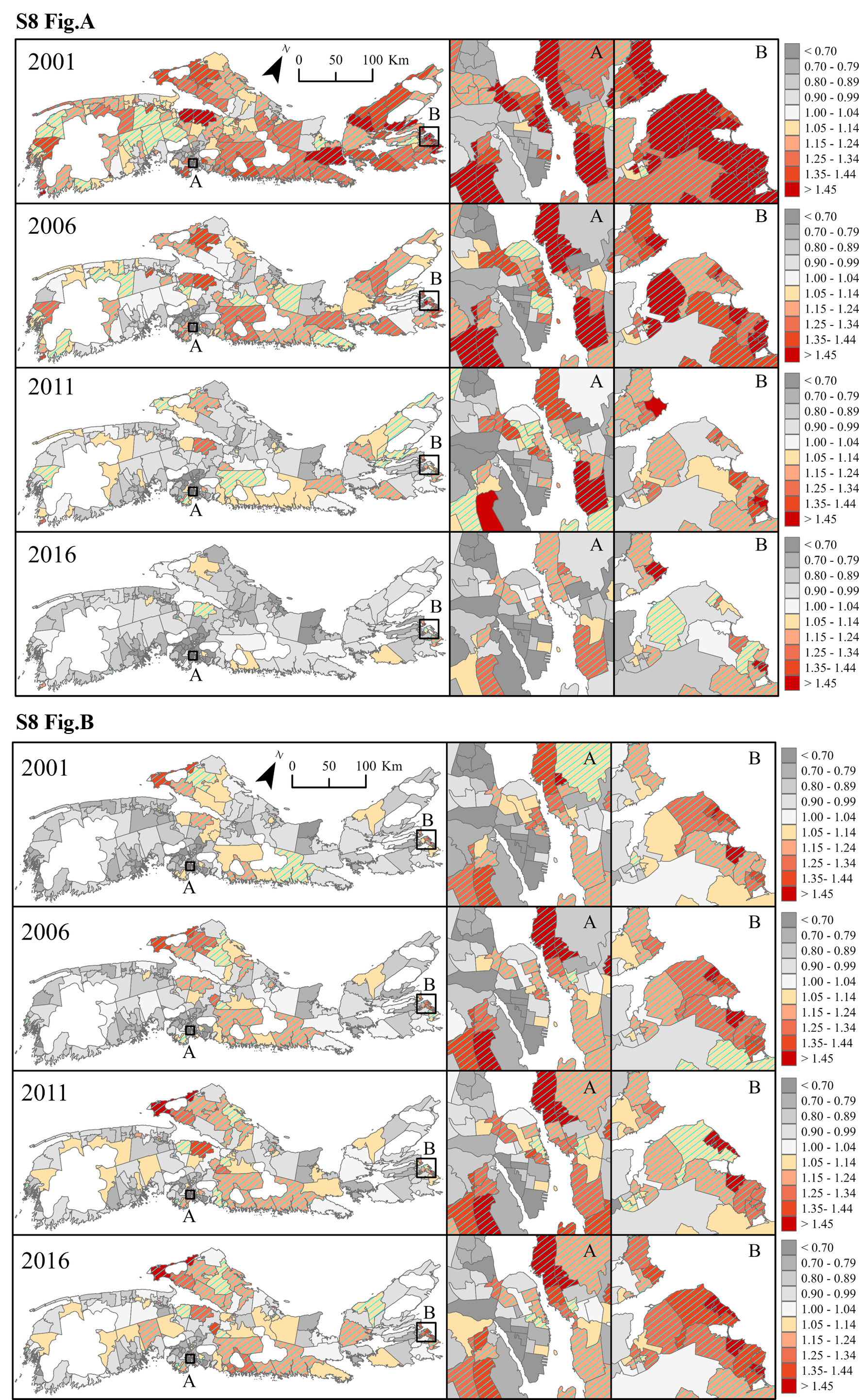

Supplement: S8 Fig — Posterior predictions displaying median relative risk (RR) with overlay of exceedance probability (Phigh ≥ 0.8) for lung cancer by time period for males (A) and females (B), Nova Scotia. Insets A and B represent the densely populated areas of Halifax and Sydney, respectively. Base Map Source: Statistics Canada, Census Dissemination Areas Boundary File, 17 Nov 2021. Reproduced and distributed on an “as is” basis with the permission of Statistics Canada [19]. (TIF) [file pone.0325523.s011.tif]

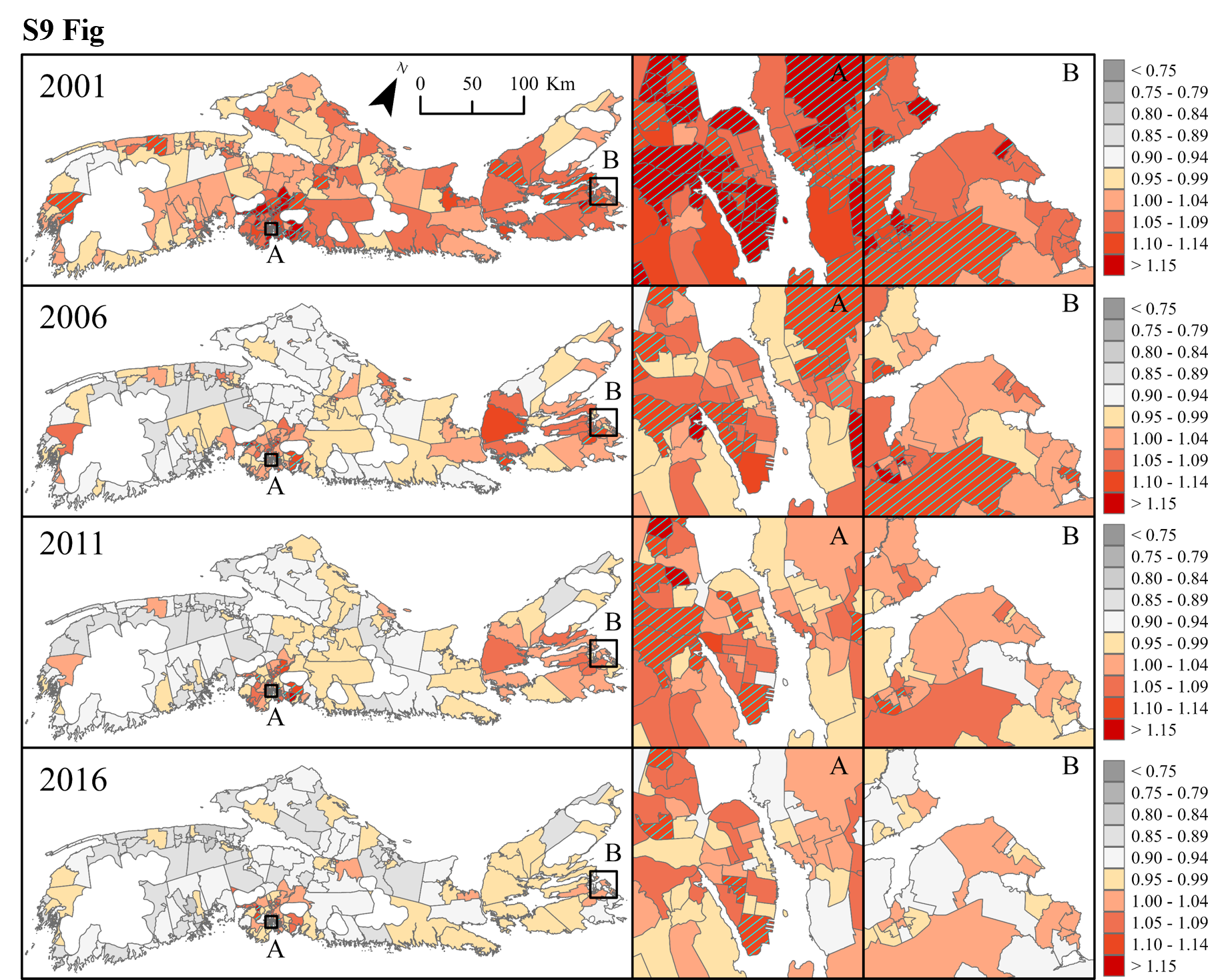

Supplement: S9 Fig — Posterior predictions displaying median relative risk (RR) with overlay of exceedance probability (Phigh ≥ 0.8) for female breast cancer by time period, Nova Scotia. Insets A and B represent the densely populated areas of Halifax and Sydney, respectively. Base Map Source: Statistics Canada, Census Dissemination Areas Boundary File, 17 Nov 2021. Reproduced and distributed on an “as is” basis with the permission of Statistics Canada [19]. (TIF) [file pone.0325523.s012.tif]

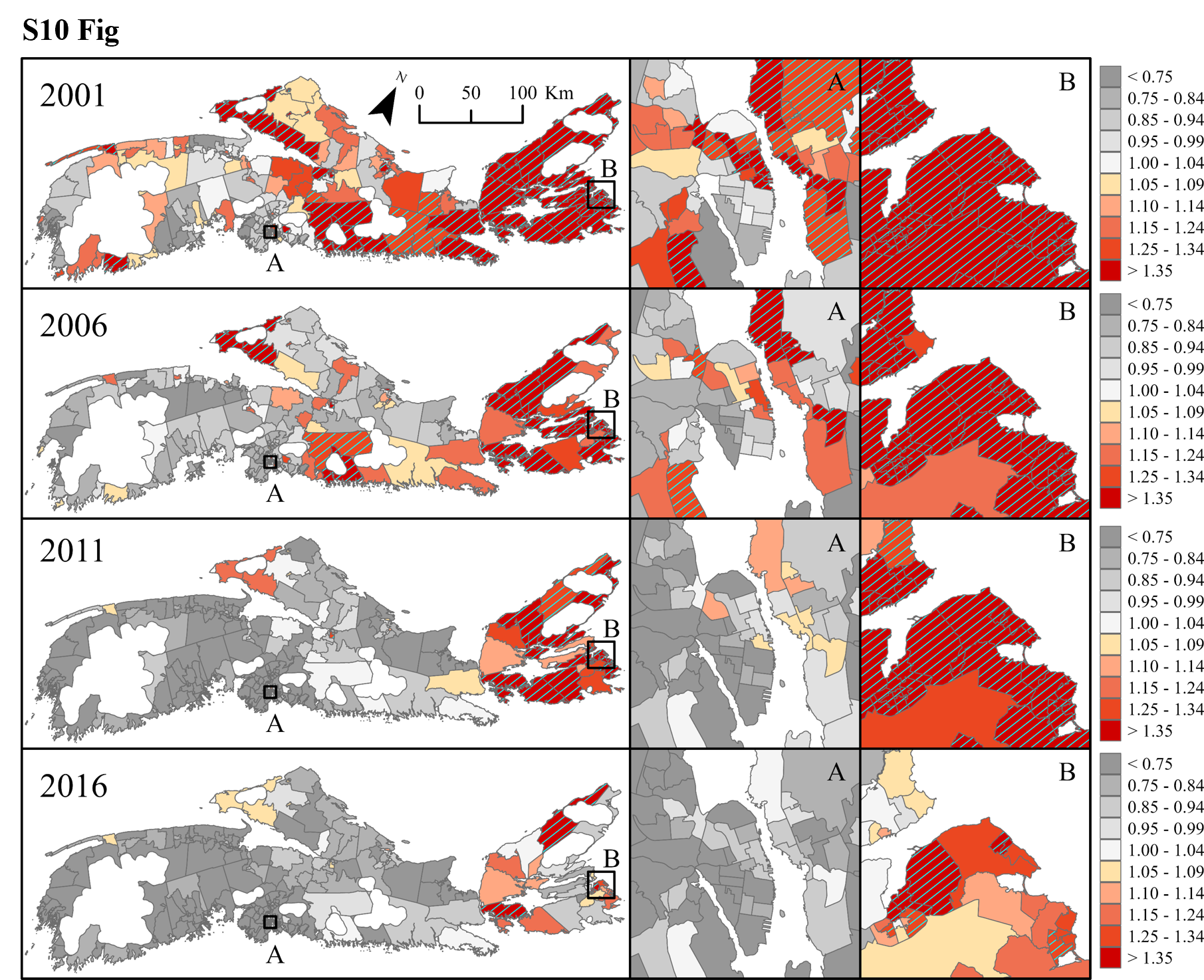

Supplement: S10 Fig — Posterior predictions displaying median relative risk (RR) with overlay of exceedance probability (Phigh ≥ 0.8) for female cervical cancer by time period, Nova Scotia. Insets A and B represent the densely populated areas of Halifax and Sydney, respectively. Base Map Source: Statistics Canada, Census Dissemination Areas Boundary File, 17 Nov 2021. Reproduced and distributed on an “as is” basis with the permission of Statistics Canada [19]. (TIF) [file pone.0325523.s013.tif]
